# Activation of transient receptor potential vanilloid 1 ameliorates tau accumulation‐induced synaptic damage and cognitive dysfunction via autophagy enhancement

**DOI:** 10.1111/cns.14432

**Published:** 2023-08-29

**Authors:** Tao Zhang, Yuan Tian, Xiaoqing Zheng, Ruomeng Li, Li Hu, Xindong Shui, Yingxue Mei, Quling Wang, Mi Zhang, Xiuzhi Zheng, Long Wang, Dongmei Chen, Wucheng Tao, Tae Ho Lee

**Affiliations:** ^1^ Fujian Key Laboratory of Translational Research in Cancer and Neurodegenerative Diseases, School of Basic Medical Sciences Fujian Medical University Fuzhou China; ^2^ Key Laboratory of Brain Aging and Neurodegenerative Diseases, School of Basic Medical Sciences Fujian Medical University Fuzhou China

**Keywords:** autophagy, capsaicin, cognition, tau, tauopathy, transient receptor potential vanilloid 1

## Abstract

**Aims:**

The autophagy‐lysosomal pathway is important for maintaining cellular proteostasis, while dysfunction of this pathway has been suggested to drive the aberrant intraneuronal accumulation of tau protein, leading to synaptic damage and cognitive impairment. Previous studies have demonstrated that the activation of transient receptor potential vanilloid 1 (TRPV1) by capsaicin has a positive impact on cognition and AD‐related biomarkers. However, the effect and mechanism of TPRV1 activation on neuronal tau homeostasis remain elusive.

**Methods:**

A mouse model of tauopathy was established by overexpressing full‐length human tau in the CA3 area. Mice were fed capsaicin diet (0.0125%) or normal diet for 9 weeks. The cognitive ability, synaptic function, tau phosphorylation levels, and autophagy markers were detected. In vitro, capsaicin‐induced alterations in cellular autophagy and tau degradation were characterized using two cell models. Besides, various inhibitors were applied to validate the role of TRPV1‐mediated autophagy enhancement in tau clearance.

**Results:**

We observed that TRPV1 activation by capsaicin effectively mitigates hippocampal tau accumulation‐induced synaptic damages, gliosis, and cognitive impairment in vivo. Capsaicin promotes the degradation of abnormally accumulated tau through enhancing autophagic function in neurons, which is dependent on TRPV1‐mediated activation of AMP‐activated protein kinase (AMPK) and subsequent inhibition of the mammalian target of rapamycin (mTOR). Blocking AMPK activation abolishes capsaicin‐induced autophagy enhancement and tau degradation in neurons.

**Conclusion:**

Our findings reveal that capsaicin‐induced TRPV1 activation confers neuroprotection by restoring neuronal tau homeostasis via modulating cellular autophagy and provides additional evidence to support the potential of TRPV1 as a therapeutic target for tauopathies.

## Graphical abstract

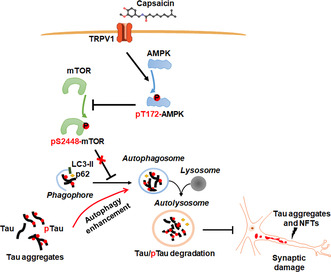

## 1 INTRODUCTION

The microtubule‐associated protein tau plays a fundamental role in modulating the normal functioning and morphology of neurons. Although tau is predominantly localized in axons of adult neurons, it has been shown that tau is evenly distributed in the soma and neurites of developing neurons.^1^ The enrichment of tau in axons highlights its critical role in regulating axonal growth and transport.^2^ The primary function of tau is to regulate microtubule dynamics by interacting with αβ‐tubulin heterodimers, thereby stabilizing microtubules.^1^ The abnormal accumulation and phosphorylation of tau have been implicated in multiple neurodegenerative diseases known as tauopathies, such as Alzheimer's disease (AD) and frontotemporal dementia.^3^ The hyperphosphorylation of tau not only causes axonal microtubule depolymerization but also triggers the formation of tau aggregates and neurofibrillary tangles (NFTs).^3^ The phosphorylation of tau has been reported to increase by at least threefold in brains of AD patients compared with those of aged controls.^4^ In addition, the missorting of tau from axons to somatodendritic compartments is another important pathological feature of AD.^5^ Although the molecular mechanisms underlying pathological tau accumulation and mislocalization in AD have not been fully elucidated, recent studies have revealed a possible involvement of tau degradation deficiency in the disruption of tau homeostasis.^6^

The clearance of tau protein is mainly regulated by the ubiquitination‐proteasome system (UPS) and the autophagy‐lysosomal system.^7^ Depending on the modification and aggregation states of tau, the UPS or autophagy pathway may be activated to degrade intracellular tau species.^8^ For example, the proteasome‐mediated degradation is likely the main pathway responsible for tau turnover under physiological conditions, while pathological tau species such as oligomers and NFTs may largely be eliminated by the autophagy pathway.^7^ In particular, studies have proven the colocalization of autophagy markers LC3 and p62 with hyperphosphorylated tau proteins in the postmortem brains of AD patients,^9^ suggesting a close association between tau clearance and the autophagy pathway. Nevertheless, the maturation of autophagolysosomes and their retrograde transport in neurons are strongly inhibited in brains of AD patients, as evidenced by the extensive accumulation of autophagic vacuoles in neurons with neurofibrillary pathology.^10^ These findings indicate that autophagic dysfunction contributes significantly to the disruption of neuronal proteostasis and the progressive accumulation of hyperphosphorylated tau proteins.^11^ Furthermore, the stimulation of autophagic function in neurons efficiently promotes tau degradation in vitro and in vivo, leading to significantly reduced tau aggregation and improved neuronal functions.^12^, ^13^, ^14^ Therefore, manipulating the autophagy pathway could be a promising strategy to target tauopathies.

Capsaicin, a pungent component of chili peppers, is a potent agonist of transient receptor potential vanilloid 1 (TRPV1). The consumption of spicy food was suggested to be associated with better cognition and lower levels of core AD biomarkers in several clinical studies.^15^, ^16^ Further animal and cellular studies showed that capsaicin is able to reduce amyloid‐β (Aβ) generation by promoting the non‐amyloidogenic processing of amyloid‐precursor proteins,^17^ while its role in modulating tau pathology in AD is less characterized. TRPV1 is a non‐selective cation channel expressed widely in a subset of peripheral sensory neurons. The major function of TRPV1 is to control calcium transmission evoked by chemical or physical nociception in primary afferent neurons.^18^ Recent studies have demonstrated that TRPV1 is also expressed in the central nervous system and is actively involved in regulating synaptic plasticity.^19^ The exact role of TRPV1 in the pathogenesis of AD remains controversial, as some studies have reported that TRPV1 activation rescues AD‐related synaptic dysfunction and cognitive deficits,^20^, ^21^ while others have shown that upregulation or activation of TRPV1 exacerbates AD pathologies by increasing Aβ and phosphorylated tau levels.^22^, ^23^ The activation of TRPV1 in microglia, the principal immune cells in the brain, was found to restore energy metabolism and promote microglia‐mediated phagocytosis and degradation of Aβ or α‐synuclein aggregates.^24^, ^25^, ^26^ However, the influence of TRPV1 activation on pathological tau phosphorylation and accumulation in neurons remains elusive.

In the present study, we aim to clarify whether and how the TRPV1 agonist capsaicin affects neuronal tau pathology, by employing cellular and animal models of tauopathy. Specifically, we characterize the impact of capsaicin treatment on cellular autophagy and the involvement of autophagy in regulating tau degradation in neurons. The molecular mechanism is dissected by applying different inhibitors. Meanwhile, the influence of TRPV1 activation on synaptic function and cognition is studied using a tauopathy mouse model.

## 2 MATERIALS AND METHODS

### 2.1 Chemicals and reagents

The TRPV1 agonist capsaicin (CYR‐L0011, Cap) was purchased from Cuiyirun Biotechnology Co., Ltd. (Sichuan, China) with a purity of 99.5%. The TRPV1 antagonist capsazepine (C191, CPZ) was purchased from Merck (Darmstadt, Germany). The recombinant adeno‐associated virus containing CMV‐hTau‐mCherry‐WPRE (PT‐1912, hTau virus) or CMV‐mCherry‐WPRE (PT‐1096, vector virus) was packaged and provided by BrainVTA (Wuhan, China). The full‐length human tau sequence (2N4R) was used in the present study. The CMV‐mCherry‐GFP‐LC3 construct (D2816) was obtained from Beyotime (Shanghai, China). The autophagy inhibitors chloroquine (HY‐17589A, CQ), 3‐methyladenine (HY‐19312, 3‐MA), and the proteasome inhibitor MG‐132 (HY‐13259) were all purchased from MCE (Shanghai, China). The AMPK inhibitor compound C (D139352, Com.C) was provided by Aladdin (Shanghai, China). Hoechst 33342 (E607328) for cell nuclear staining was obtained from Sangon Biotech (Shanghai, China). Human TRPV1 SiRNA (SC‐36826) and control SiRNA (SC‐37007) were purchased from Santa Cruz Biotechnology (Texas, USA).

### 2.2 Animals, stereotaxic surgery, and treatment

Seven‐week‐old male C57BL/6 mice were purchased from SLAC Laboratory Animal Co., Ltd. (Shanghai, China), and maintained in a standard specific pathogen‐free area at the animal facility of Fujian Medical University on a 12 h light–dark cycle and provided free access to food and water. After 1 week of habituation, mice were subjected to stereotaxic surgery for viral infusion, as previously described.^27^ In brief, mice were anesthetized by isoflurane inhalation and fixed in the stereotaxic apparatus. After iodophor sterilization, an incision was made along the anteroposterior axis to expose the cranium. For stereotaxic injection, an injection hole was made with a handheld drill at AP −2.2 mm, ML −2.7 mm, and DV −2.3 mm relative to the bregma. The hTau virus or vector virus (5.4 × 10^12^ viral genomes/mL, total volume of 700 nL) was infused to the CA3 area by using a microsyringe pump (Kd Scientific) at a rate of 125 nL/min. The needle was kept in place for 5 min before withdrawal. The mouse was then sutured and placed on a heating pad for recovery. One week after the surgery, mice were divided into four groups and were treated with either normal diet or capsaicin diet (0.0125%) for 9 weeks. Body weight was measured once a week. All animal experiments were reviewed and approved by the Animal Welfare & Ethics Committee of Fujian Medical University (IACUC FJMU 2022‐Y‐0547).

### 2.3 Morris water maze

After the treatment, all mice were subjected to Morris water maze to evaluate the spatial learning and memory according to a previous study.^28^ Mice were transferred to the behavioral test room at least 30 min prior to the experiment. In general, mice were first trained in a visible platform test (day one) and a hidden platform test (day two to five) consecutively, with five trials per day (trial interval ~ 20 min) in the water maze equipped with a camera (RWD). The cut‐off time for each trial was set to 60 s. If a mouse reached the platform within 60 s, it was allowed to stay on the platform for 5 s. Otherwise, the mouse was led to the platform and allowed to stay for 20 s before being returned to its home cage. On day 6, the probe trial was performed, the platform was removed, and mice were allowed to freely swim in water for 60 s. In each trial, the mouse was gently placed in the water from the starting position, facing the wall of the water maze. The data were analyzed using the Smart program (Version 3.0.06) provided by Panlab Harvard Apparatus (Barcelona, Spain).

### 2.4 Cell culture, transfection, and treatment

293 cells stably overexpressing human 2N4R tau (293‐hTau) were maintained as described in our previous study.^29^ Mouse primary neurons were isolated and cultured according to our previous protocol,^30^ and neurons on seventh day in vitro were used for compound treatment. TurboFect transfection reagent (R0533; Thermo Fisher) was used for plasmid or SiRNA transfection based on the manufacturer's introduction. Cells were treated with 1 μM or 10 μM capsaicin for 24 h before sample collection for the analysis of total tau, phosphorylated tau, and autophagy markers. For the measurement of autophagic flux, cells were first treated with 10 μM capsaicin for 20 h, and then CQ was added to the medium at a concentration of 5 μM. Samples were collected after 4 h of co‐incubation. To identify which pathway was involved in tau level regulation, we used the autophagy inhibitors CQ and 3‐MA and the proteasome inhibitor MG132. For CQ treatment, cells were first incubated with 10 μM of capsaicin for 15 h, and then CQ at 20 μM was added to the medium for another 9 h. For 3‐MA and MG132 treatment, the compounds were added to cell media at 5 mM and 10 μM after 12 h of capsaicin pre‐incubation, respectively. To block the TRPV1 activation, CPZ (10 μM) together with capsaicin was added to cells at the beginning of incubation. Com.C (20 μM) was applied to the cells half an hour prior to sample collection. The total incubation time for all cell experiments was set to 24 h.

### 2.5 Immunofluorescence

Coronal sections (4 μm) were made from paraffin‐embedded brain tissues for immunofluorescence. After deparaffinization, rehydration, and antigen retrieval, sections were blocked in goat serum (AR0009; Boster Bio) at room temperature for 1 h. Cells seeded on coverslips were rinsed with cold PBS three times and were fixed using 4% paraformaldehyde for 30 min at room temperature. Then, cells were permeabilized in PBS with 0.5% Triton‐X100 for 5 min, and subsequently blocked in PBS containing 7% FBS for 1 h at room temperature.^31^ Primary antibodies diluted in the blocking buffer were then applied to samples and were incubated at 4°C overnight. After three rinses in PBS containing 0.1% Tween‐20, Alexa Fluor 488‐ or 546‐conjugated secondary antibodies (Invitrogen) were added to the samples and were incubated at room temperature for 1 h. After washing three times in PBS, samples were further stained with Hoechst 33342 for 5 min. Finally, all samples were rinsed with water and mounted onto slides using antifade medium (0100‐35; SouthernBiotech). Samples were imaged using a confocal microscope (Leica SP5) or a conventional fluorescence microscope (Zeiss Axio Imager 2). The images were analyzed using ImageJ software (Version 1.50i; NIH).

### 2.6 Golgi‐Cox staining

Golgi‐cox staining was carried out based on the manufacturer's instructions using the FD Rapid GolgiStain™ Kit (PK401). After thorough brain tissue impregnation, sections (200 μm) were prepared by a vibratome (Leica). The sections were then stained and imaged using a Zeiss primo star microscope equipped with a 100× oil objective. The dendritic spines were counted using ImageJ software.

### 2.7 Immunoblot analysis

Immunoblot analyses were performed according to our previous report.^32^ Total proteins were extracted from mouse hippocampus or cells in radioimmunoprecipitation assay (RIPA) buffer with protease and phosphatase inhibitors. The protein concentration was determined using a BCA protein assay kit (Beyotime). Protein samples (15–30 μg) were separated by SDS/PAGE and transferred to polyvinylidene fluoride membranes (Merck) by semidry transfer (Bio‐Rad). Membranes were then blocked with 5% BSA‐TBST or 5% milk‐TBST at room temperature for 1 h, prior to incubation with primary antibodies at 4°C overnight. After washing the membrane with TBST three times to remove extra primary antibodies, HRP‐conjugated secondary antibodies were added and samples were incubated at room temperature for 1 h. Afterward, all membranes were developed using ECL chemiluminescent HRP substrate (Merck) and imaged with a Chemidoc imaging system from Bio‐Rad. Information about antibodies used in the study are available in Table S1. The protein levels were quantified based on the corresponding loading control by ImageJ software, and the relative protein contents in all groups were further compared by normalizing to the control group.

### 2.8 Statistical analysis

All data are expressed as mean ± standard error (SD), and statistical analyses were carried out using GraphPad Prism software (Version 8.3.0). The data were first checked by the Shapiro–Wilk normality test. Differences between two groups were determined out by two‐tailed unpaired *t*‐test, and multiple comparisons were performed using one‐way analysis of variance (ANOVA) followed by Tukey's *post‐hoc* test. Data that did not exhibit normal distribution were analyzed via nonparametric tests. The group size and statistical tests were indicated in the figure legends. A *p* value <0.05 was considered statistically significant.

## 3 RESULTS

### 3.1 Capsaicin treatment ameliorates cognitive impairments in a mouse model of tauopathy

**FIGURE 1:**
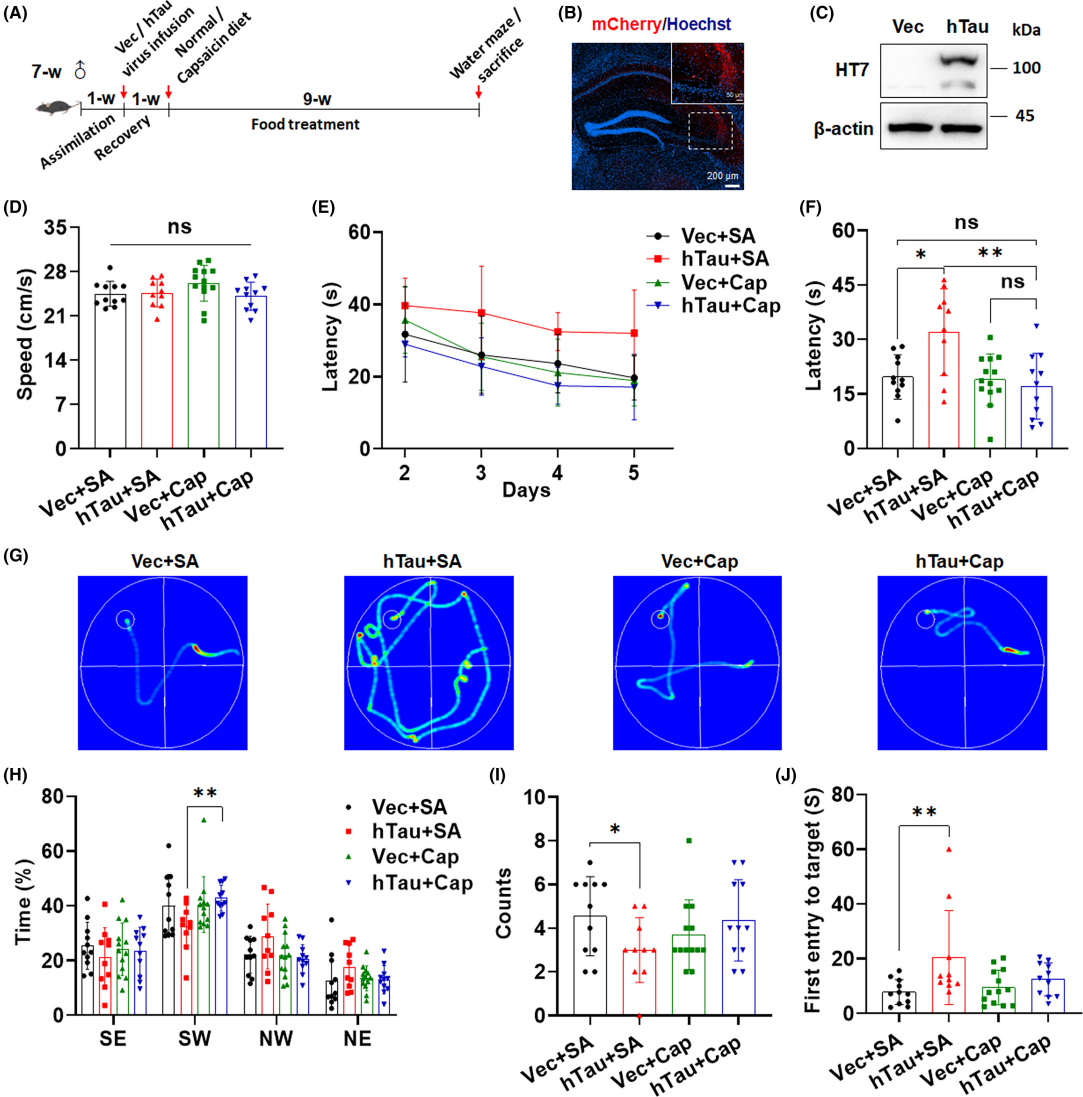
Capsaicin treatment ameliorates cognitive impairments in a mouse model of tauopathy. (A) Experimental paradigm of the in vivo study. (B,C) Validation of the expression of mCherry‐hTau in the CA3 area of the mouse hippocampus at 1 week after stereotaxic infusion of the hTau virus using immunofluorescence (B) and immunoblot analysis (C). (D–G) The Morris water maze (MWM) was used to assess the effect of capsaicin treatment on the spatial learning and memory of mice injected with vector or hTau virus. The mean swimming speed (D), escape latency in the training phase (E), the escape latency (F), and representative swim trajectories on day five (G) are shown. (H–J) The percentage of time spent in different quadrants (H), the counts of platform crossings (I), and the time of the first entry to the platform (J) for all four groups of mice in the probe trial (day six). n = 10–13 mice/group. *p < 0.05, **p < 0.01, ns, not significant. One‐way ANOVA followed by Tukey's *post‐hoc* test in D and F. Two‐tailed unpaired *t*‐test in H and I. Mann–Whitney nonparametric test in J.

Tau hyperphosphorylation and accumulation are early pathological changes in AD and are highly correlated with cognitive dysfunction during disease progression. We first established a mouse model of tauopathy by stereotaxic injection of adeno‐associated virus expressing hTau‐mCherry (hTau virus) into the CA3 area of hippocampus, which is known to cause synaptic dysfunction and cognitive impairments.^27^ After 1 week of recovery, mice were divided into four groups based on the treatment regimen and were fed a normal diet or capsaicin‐containing diet (0.0125%) for 9 weeks (Figure 1A). Body weight of all mice increased progressively during the treatment period (Figure S1), indicating that the concentration of capsaicin was safe and tolerable in mice. The expression of hTau‐mCherry in CA3 was validated by detecting mCherry signals using immunofluorescence and by checking hippocampal hTau‐mCherry protein levels via a human tau‐specific HT7 antibody (Figure 1B,C). To determine whether capsaicin treatment ameliorates hTau overexpression‐induced cognitive dysfunction, we performed the Morris water maze (MWM) test to evaluate spatial learning and memory after treatment. The swimming speed in the visible platform test was comparable among the four groups (Figure 1D). In the hidden platform test (day two to five), the escape latency declined gradually as training progressed, while the hTau mice treated with normal diet (hTau + SA) showed a significantly longer latency than mice in the control group (Vec + SA), suggesting impaired cognition by hTau overexpression (Figure 1E–G). However, the escape latency of hTau mice treated with the capsaicin diet (hTau + Cap) was similar to that of the vector groups on day five and was much shorter than that of the hTau + SA mice (Figure 1E–G). As shown in the probe trial (Figure 1H), all groups had a preference for the SW quadrant where the platform was positioned; however, the percentage of time spent in target quadrant for the hTau + SA group was significantly lower than that of the hTau + Cap group (Figure 1H). We also observed that the hTau + SA group exhibited fewer target crossings and a longer latency to reach the platform than the Vec + SA group (Figure 1I,J), and that capsaicin treatment increased the average number of target crossings in hTau‐expressing mice but reduced the average time of first entry to the platform (Figure 1I,J), although the differences were not significant. The data demonstrate that capsaicin treatment is able to improve the cognitive ability of mice with tau overexpression in the hippocampus.

### 3.2 Capsaicin treatment attenuates abnormal tau accumulation and phosphorylation in the hippocampus

**FIGURE 2:**
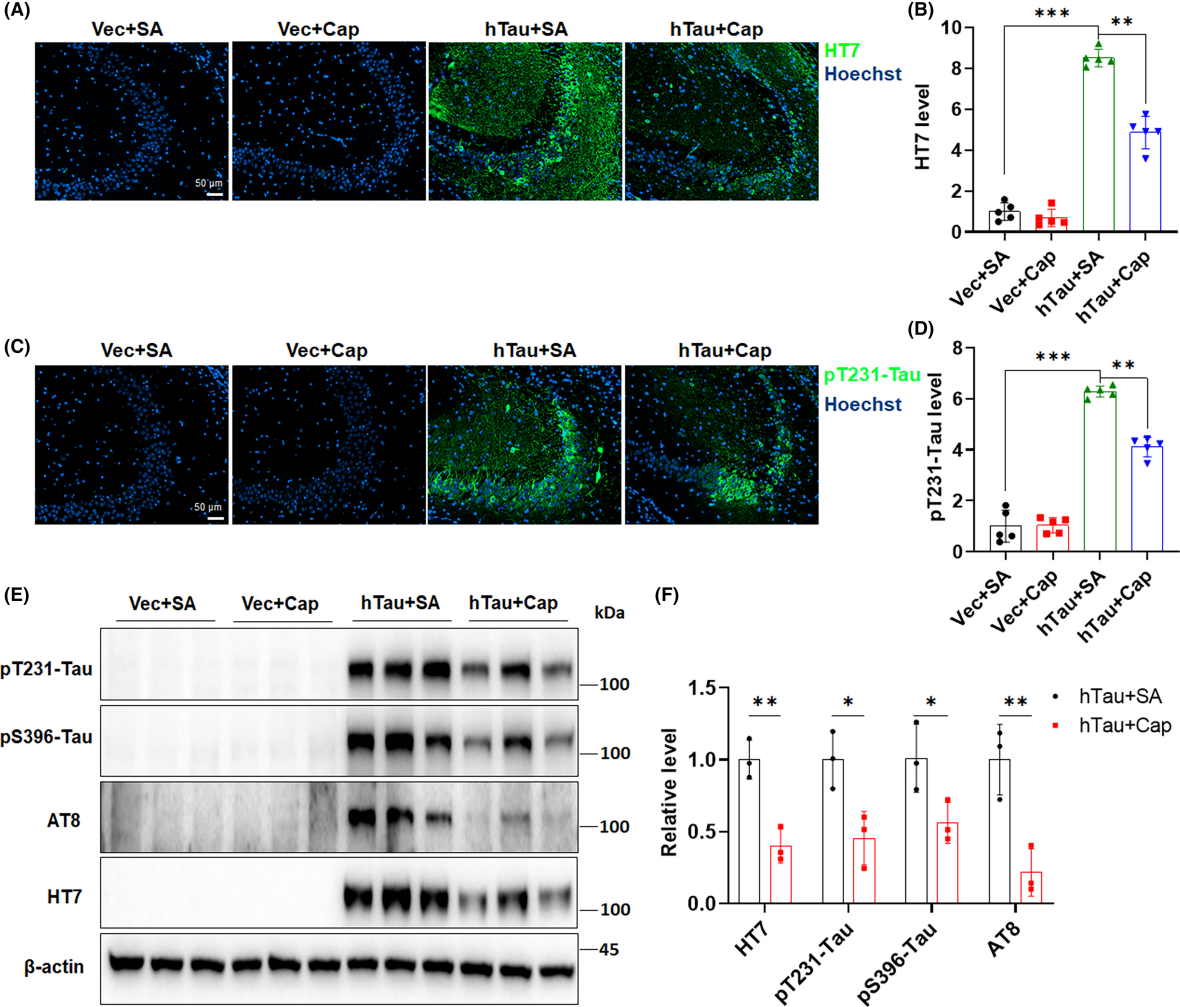
Capsaicin treatment attenuates abnormal tau accumulation and phosphorylation in the hippocampus. (A,B) Characterization of the level of mCherry‐hTau in the CA3 area of mice treated with or without capsaicin for 9 weeks using the HT7 antibody via immunofluorescence. (C,D) Analysis of the level of tau phosphorylation at Thr231 (pT231‐Tau) in the CA3 area of mice treated with or without capsaicin for 9 weeks via immunofluorescence. (E,F) Determination of the level of total mCherry‐hTau, and phosphorylated hTau at Thr231 (pT231), Ser396 (pS396), and Ser202/Thr205 (AT8), respectively, in the hippocampus of mice treated with or without capsaicin using immunoblot analysis. *p < 0.05, **p < 0.01, ***p < 0.001. One‐way ANOVA followed by Tukey's *post‐hoc* test in B and D, and two‐tailed unpaired *t*‐test in F.

To characterize whether capsaicin treatment can alleviate tau pathology in the hippocampus, we examined levels of total hTau and phosphorylated tau at multiple AD‐related sites. We first found that the strong HT7 signal in CA3 areas of hTau mice was dramatically reduced by capsaicin treatment (Figure 2A,B). Tau phosphorylation at Thr231 (pT231‐Tau) is important for its microtubule binding and has been proven to increase in the preclinical stage of AD.^33^, ^34^ Our data showed that the accumulation of pT231‐Tau in the CA3 area was markedly attenuated by capsaicin treatment (Figure 2C,D). In line with the immunofluorescence data, we also observed that the level of total hTau, as well as tau phosphorylation at Thr231 (pT231), Ser396 (pS396), and Ser202/Thr205 (AT8), was substantially decreased in the hippocampus of hTau mice fed with capsaicin diet, when compared with that of mice in the hTau + SA group (Figure 2E,F). These results demonstrate that capsaicin treatment potently prevents the accumulation of total and phosphorylated tau proteins in the hippocampus of the model mice.

### 3.3 Capsaicin treatment ameliorates abnormal tau accumulation‐induced synaptic dysfunction and gliosis in the hippocampus

**FIGURE 3:**
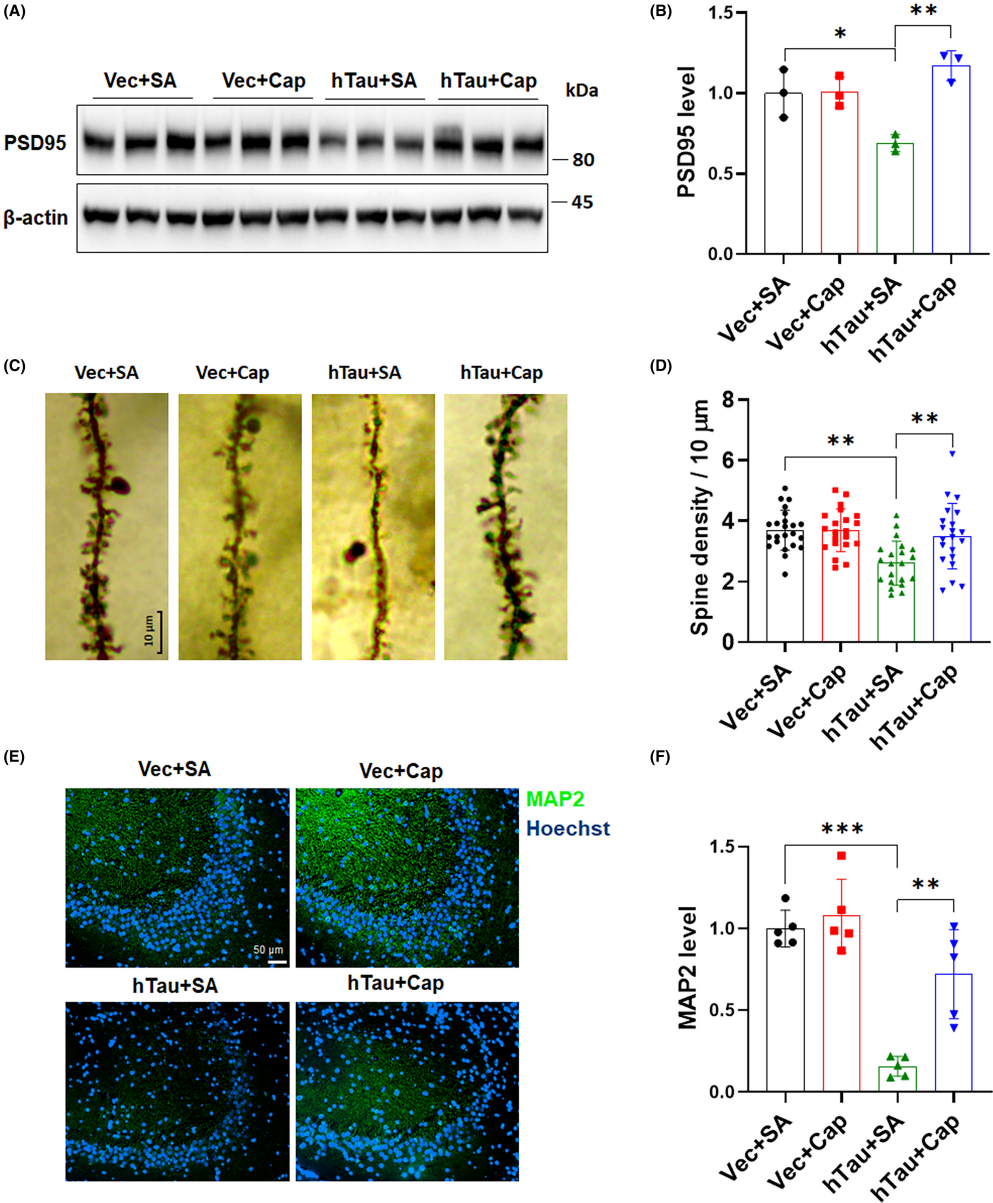
Capsaicin treatment alleviates abnormal tau accumulation‐induced synaptic dysfunction in the hippocampus. (A,B) Evaluation of the PSD95 level in the hippocampus of mice treated with or without capsaicin for 9 weeks by immunoblot analysis. (C,D) Analysis of the number of dendritic spines in the CA3 area of hippocampus in mice treated with or without capsaicin for 9 weeks using the Golgi‐Cox staining. Representative images of dendritic spines are shown. Data from 21 to 23 randomly selected dendrites in the CA3 area of three mice per group were averaged. (E,F) Immunofluorescence staining of MAP2 in the CA3 area of hippocampus in mice treated with or without capsaicin for nine weeks. *p < 0.05, **p < 0.01, ***p < 0.001. One‐way ANOVA followed by Tukey's *post‐hoc* test in B, D, and F.

Pathological tau affects synaptic functions by interfering with the formation of postsynaptic densities and dendritic spines.^35^ To determine whether capsaicin treatment rescues the hippocampal synaptic dysfunction induced by tau overexpression, we first measured the protein level of postsynaptic density protein‐95 (PSD95) in mouse hippocampi and observed that overexpression of hTau indeed downregulated the expression of PSD95 in the hippocampus, while this downregulation was reversed by capsaicin treatment (Figure 3A,B). As PSD95 is critically involved in regulating the stability and dynamics of dendritic spines where the majority of excitatory synapses are located,^36^ we sought to understand whether changes in PSD95 protein levels lead to any morphological alteration in dendrites and dendritic spines in the hippocampus. Golgi‐Cox staining revealed that mice in the hTau + SA group manifested a significantly lower dendritic spine density than those in the Vec + SA group (Figure 3C,D), implicating a loss of hippocampal dendritic spines after tau overexpression. However, the dendritic spine loss in hTau mice was recovered by capsaicin treatment (Figure 3C,D), as demonstrated by a robust increase in the spine density in the hTau + Cap group. This restoration of PSD95 expression and the dendritic spine density consistently suggest that capsaicin treatment protects neurons from abnormal tau accumulation‐induced dendritic injury. The mislocalization of tau to dendrites following its overexpression may damage the structure of microtubules and accelerate dendritic degeneration during disease progression.^37^ To further characterize the impact of capsaicin on tau accumulation‐induced synaptic damage, we examined the expression of the somatodendritic marker microtubule‐associated protein 2 (MAP2) in mouse hippocampus. As shown in Figure 3E,F, tau overexpression decreased the average MAP2 fluorescence signal in CA3 areas of hTau + SA mice. However, capsaicin treatment was able to restore MAP2 expression in the hippocampus of hTau mice. Together, these findings confirm that capsaicin treatment efficiently counteracts synaptic dysfunctions caused by tau accumulation through normalizing PSD95 expression and preserving dendritic spines.

**FIGURE 4:**
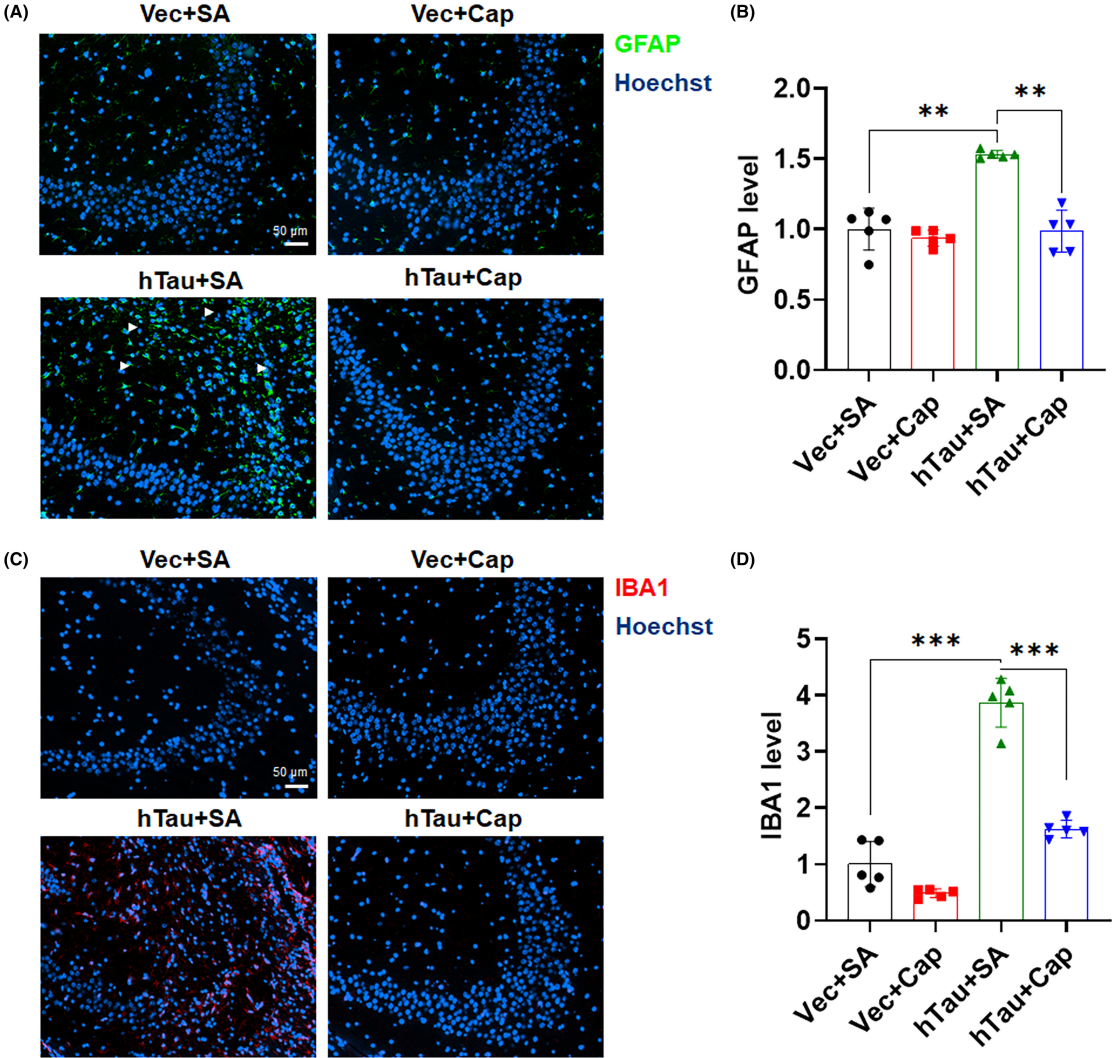
Capsaicin treatment suppresses abnormal tau accumulation‐induced gliosis in the hippocampus. (A,B) Detection of astrocytes activation by GFAP staining in the CA3 area of hippocampus in mice treated with or without capsaicin for 9 weeks. The arrowheads indicate representative GFAP signals. (C,D) Analysis of the microglia activation by staining of IBA1 in the CA3 area of hippocampus in mice treated with or without capsaicin for 9 weeks. **p < 0.01, ***p < 0.001. One‐way ANOVA followed by Tukey's *post‐hoc* test.

In addition to extensive synaptic injury, tau accumulation and hyperphosphorylation are also connected with glial activation and the neuroinflammatory response in the brain.^38^ For example, tau oligomers have been found to co‐localize with glial cells and stimulate the generation of cytokines.^39^ Glial cell activation in turn exacerbates tau pathology by enhancing tau hyperphosphorylation and facilitating its toxic propagation.^40^, ^41^ To address whether capsaicin treatment also attenuates tau overexpression‐induced glial cell activation, we assessed the expression of glial fibrillary acidic protein (GFAP) and ionized calcium‐binding adaptor molecule 1 (IBA1), markers for astrocytes and reactive microglia, respectively. The significant increase in GFAP (Figure 4A,B) and IBA1 (Figure 4C,D) expression in the hippocampus of hTau + SA mice reflected the activation of both astrocytes and microglia by tau overexpression. However, capsaicin treatment was able to suppress tau accumulation‐induced astrocyte and microglial activation, as shown by normalized GFAP and IBA1 expression in the CA3 area of hTau + Cap group (Figure 4). Therefore, capsaicin is capable of alleviating abnormal tau accumulation‐induced neuroinflammatory response in the hippocampus.

### 3.4 The AMPK/mTOR pathway mediates capsaicin treatment‐induced autophagy enhancement in the mouse hippocampus

**FIGURE 5:**
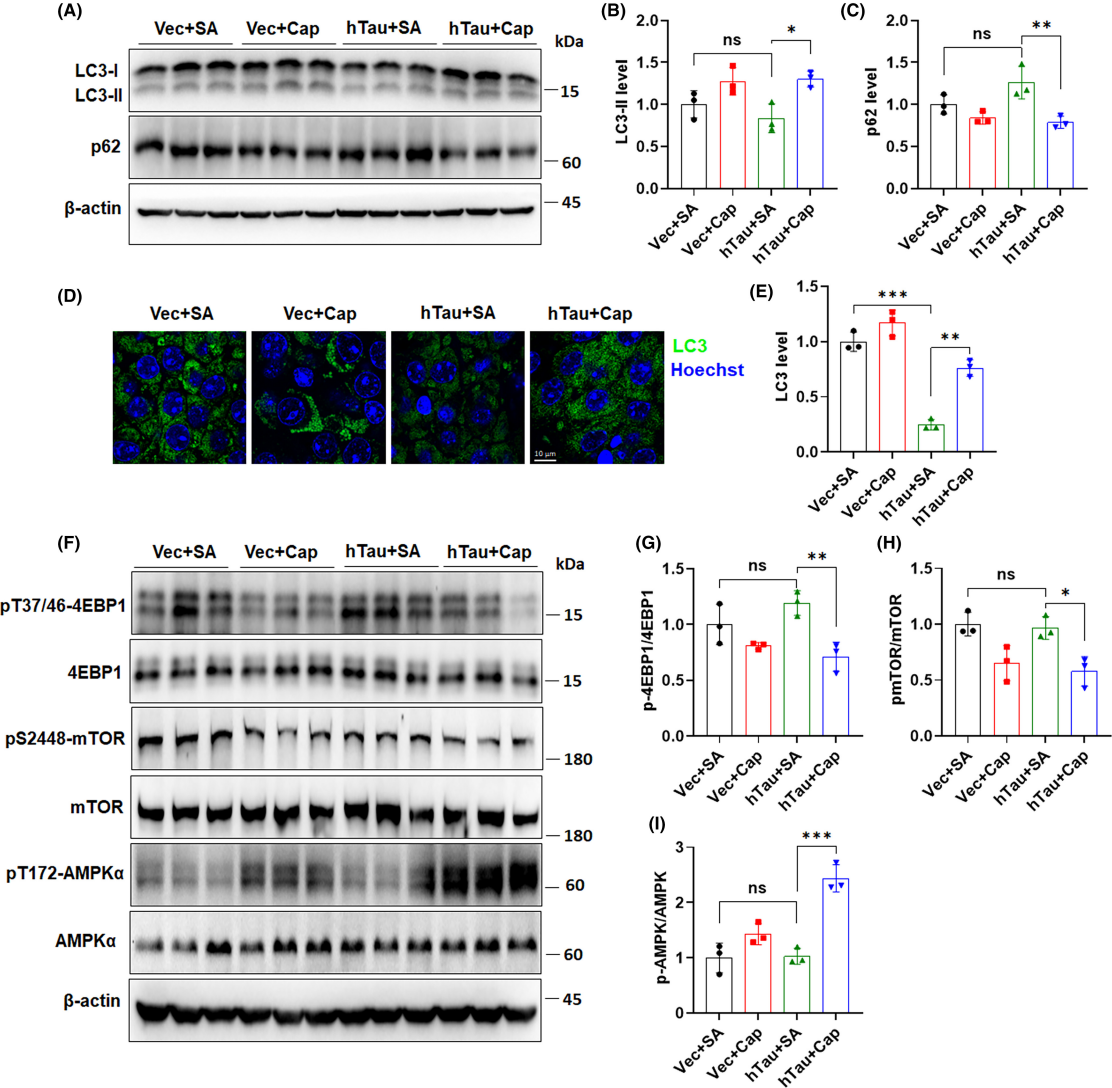
The AMPK/mTOR pathway mediates capsaicin treatment‐induced autophagy enhancement in mouse hippocampus. (A–C) Determination of levels of the autophagic markers LC3 and p62 in the hippocampus of mice treated with or without capsaicin for 9 weeks by immunoblot analysis. (D,E) Confirmation of the alteration in autophagic function by immunofluorescence staining of LC3 in the CA3 area of mouse hippocampus. (F–I) Examination of the change in AMPK/mTOR pathway activity in the hippocampus of mice receiving saline or capsaicin treatment for 9 weeks by assessing the phosphorylation of AMPK, mTOR, and 4EBP1. *p < 0.05, **p < 0.01, ***p < 0.001, ns, not significant. One‐way ANOVA followed by Tukey's *post‐hoc* test.

Our results have shown that capsaicin treatment exerts diverse neuroprotective effects in hTau mouse models, likely via downregulating neuronal tau levels. We therefore wondered if the degradation of tau in neurons is affected by capsaicin treatment. Considering the presence of autophagic dysfunction in brains of tauopathy patients and the role of TRPV1 activation in modulating the autophagy pathway,^24^, ^42^ we first examined changes in LC3 and p62 protein levels in mouse hippocampi to investigate whether the autophagy process is altered by capsaicin. It should be noted that, although we observed a decrease in LC3‐II level and an increase in p62 content in whole hippocampal lysates of hTau + SA mice compared with those of the Vec + SA mice, the difference was not statistically significant (Figure 5A–C). This might have been due to the confined expression of hTau in CA3 areas in our mouse model. To better illustrate the induction of autophagic dysfunction by tau overexpression, we measured LC3 expression in CA3 areas by confocal microscopy and found that the LC3 level in hTau + SA mice was significantly lower than that in the Vec + SA mice (Figure 5D,E), which was in accordance with a previous report showing that overexpressing tau reduces LC3‐II levels and the number of LC3 puncta.^43^ The alterations in LC3‐II and p62 levels point to autophagic dysfunction in mouse hippocampus as a result of abnormal tau accumulation. Capsaicin treatment, however, successfully reversed the expression of LC3‐II and p62 in hTau mice by significantly elevating LC3‐II and diminishing p62 in the hippocampus (Figure 5A–C). In parallel, the total LC3 level in the CA3 area was also restored in hTau mice after capsaicin administration (Figure 5D,E). The data prove that capsaicin overcomes autophagic deficiency in the brain caused by aberrant tau accumulation.

As one of the most important pathways in modulating the autophagy process, mTOR is vital for energy metabolism, neuronal growth, and cellular homeostasis.^44^ The mTOR complex 1 (mTORC1) prohibits the initiation of autophagy by blocking the interaction between autophagy‐related protein 13 and Unc‐51 like kinase 1/2 and abolishes the formation of autophagosomes.^44^ Importantly, mTORC1 activation has been documented in brains of AD patients.^43^ We evaluated the activity of mTOR in the mouse hippocampus by measuring the phosphorylation of eukaryotic translation initiation factor 4E‐binding protein 1 (4EBP1) and endogenous mTOR phosphorylation at Ser2448. We did not observe a significant change in 4EBP1 phosphorylation at T37/46 (pT37/46‐4EBP1) or mTOR phosphorylation at Ser2448 (pS2448‐mTOR) in whole hippocampal lysates (Figure 5F–H) between the hTau + SA and Vec + SA groups. Nevertheless, we noticed an obvious decline in levels of pT37/46‐4EBP1 and pS2448‐mTOR in the hTau + Cap group in comparison with the hTau + SA group, implicating that mTOR activity was inhibited by capsaicin in the hippocampus (Figure 5F–H). AMP‐activated protein kinase (AMPK), a key energy sensor in the cell, can inhibit mTOR function.^44^ Besides, TRPV1 activation by capsaicin can stimulate microglial energy metabolism and facilitate phagocytosis of misfolded proteins.^26^ We found that mice treated with capsaicin had higher levels of AMPK phosphorylation at Thr172 (pT172‐AMPK) than those without capsaicin treatment (Figure 5F,I). In particular, the hTau + Cap mice showed the highest pT172‐AMPK (Figure 5I), which implies that capsaicin indeed activates AMPK in the hippocampus. Based on these findings, we hypothesize that capsaicin modulates the AMPK/mTOR pathway and promotes cellular autophagic function, whereby neurons eliminate aberrant tau proteins in the hippocampus. The removal of tau proteins as a consequence of capsaicin‐mediated autophagy enhancement results in synaptic protection and cognitive improvement.

### 3.5 In vitro validation of autophagy enhancement by capsaicin treatment using cell models

**FIGURE 6:**
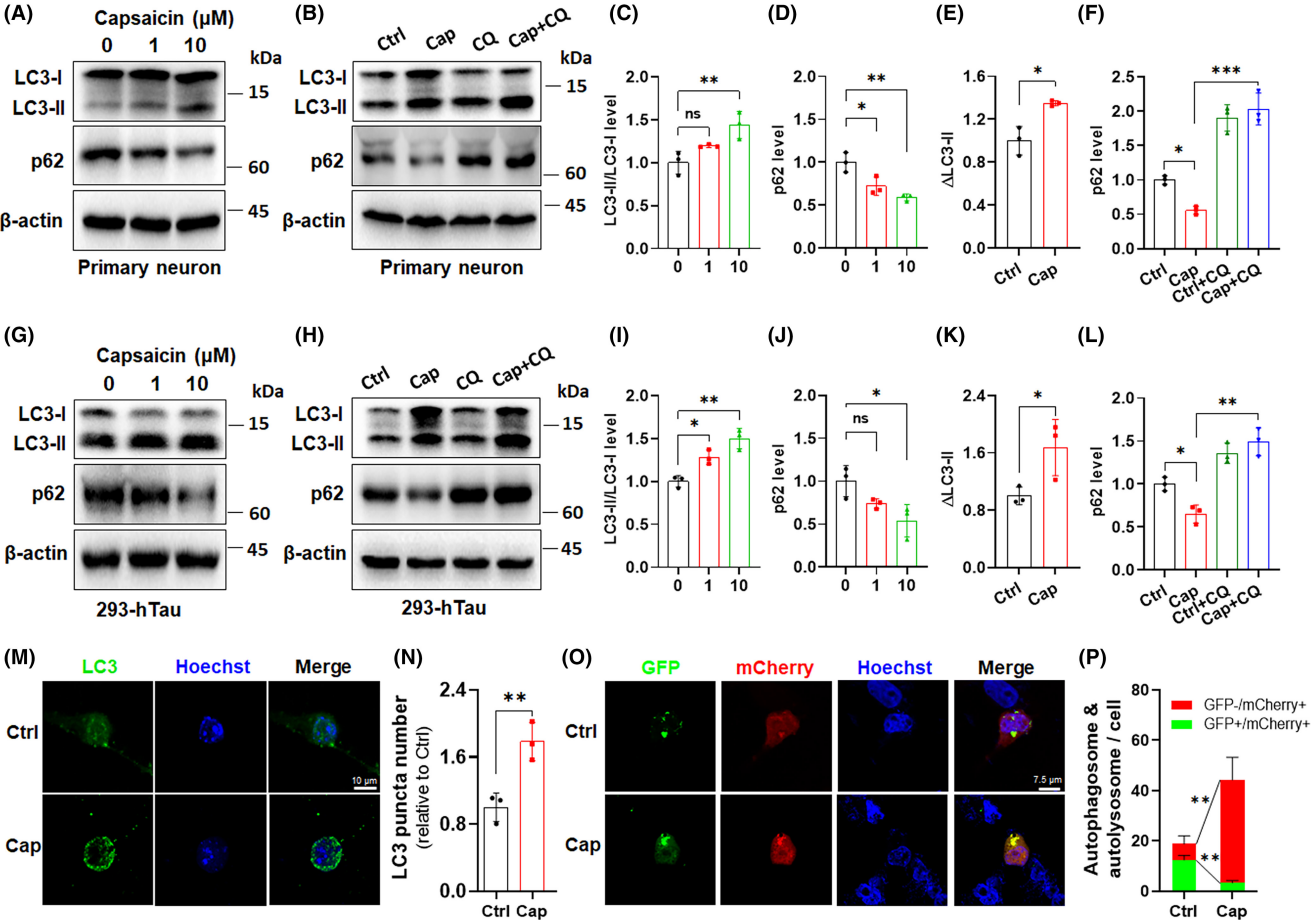
In vitro validation of autophagy enhancement by capsaicin treatment using cell models. (A–E) Characterization of the alteration in the expression of autophagy markers LC3‐II/LC3‐I (A,C) and p62 (A,D) in mouse primary neurons treated with different concentrations of capsaicin for 24 h, and determination of autophagic flux by measuring the change in LC3‐II levels in the presence of chloroquine (CQ) (B,E) in primary neurons. The expression of p62 was also detected (B,F). (G–J) Evaluation of levels of LC3‐II/LC3‐I (G,I) and p62 (G,J), and autophagic flux (H,K) in 293‐hTau cells incubated with capsaicin. The change in p62 level was also determined (H,L). (M,N) Immunofluorescence staining of LC3 puncta in 293‐hTau cells treated with vehicle (Ctrl) or capsaicin (Cap) for 24 h. (O,P) Analysis of the formation of autophagosomes and autolysosomes in 293‐hTau cells transfected with CMV‐mCherry‐GFP‐LC3 plasmids and subsequently treated with vehicle (Ctrl) and capsaicin (Cap) for 24 h by quantifying GFP and mCherry fluorescence signals. *p < 0.05, **p < 0.01, ***p < 0.001, ns, not significant. One‐way ANOVA followed by Tukey's *post‐hoc* test in C–F and I–L. Two‐tailed unpaired *t*‐test in N and P.

To further explore potential mechanisms by which capsaicin regulates cellular autophagy, we carried out in vitro experiments using two cell models. Mouse primary neurons or 293 cells stably overexpressing human tau protein (293‐hTau) were treated with 1 μM or 10 μM capsaicin for 24 h. We observed that capsaicin induced a concentration‐dependent increase in the LC3‐II/LC3‐I ratio in primary neurons. Additionally, the p62 protein level in primary neurons was decreased by capsaicin in a dose‐dependent manner (Figure 6A,C,D). These changes suggested that capsaicin positively affects cellular autophagy. To further confirm this, we determined the autophagic flux by treating primary neurons with chloroquine (CQ), an inhibitor of lysosomal degradation. CQ incubation alone upregulated LC3‐II levels in primary neurons compared with the vehicle control, while co‐treatment with capsaicin and CQ further increased the accumulation of cellular LC3‐II (Figure 6B,E). Capsaicin‐induced p62 reduction was also reversed by CQ in neurons (Figure 6B,F). Analysis of LC3‐II turnover, which is quantified by the difference in LC3‐II levels between samples incubated with and without CQ (ΔLC3‐II), demonstrated that capsaicin increased neuronal autophagic flux by about 40% (Figure 6B,E). Similarly, in 293‐hTau cells, capsaicin treatment accelerated the formation of LC3‐II and reduced p62 levels (Figure 6G,I,J). The CQ treatment further revealed enhanced LC3‐II turnover and p62 degradation caused by capsaicin in 293‐hTau cells (Figure 6H,K,L). In agreement with this, we found that 293‐hTau cells incubated with capsaicin had more LC3‐positive puncta than cells treated with vehicle (Figure 6M,N), suggesting the formation of autophagosomes upon capsaicin treatment. Moreover, we used a dual‐labeled LC3 reporting system, mCherry‐GFP‐LC3, to monitor the maturation of autolysosomes. In this system, autophagosomes retain both the GFP and mCherry fluorescence in neutral pH and will show yellow signals (GFP+/mCherry+), whereas autolysosomes strongly abrogate GFP fluorescence due to the acidic pH and mainly display red signal (GFP‐/mCherry+).^45^ The control 293‐hTau cells retained high yellow signals in the cytoplasm, corresponding to unfused autophagosomes (Figure 6O,P), while the capsaicin‐treated cells showed reduced green fluorescence but increased red/green signal ratio in the cytoplasm, indicating the maturation of autophagosomes to autolysosomes (Figure 6O,P). Thus, the in vitro data from two different cell models corroborated that capsaicin has the capability to activate cellular autophagy.

### 3.6 Capsaicin treatment‐induced tau degradation is dependent on the autophagy pathway

**FIGURE 7:**
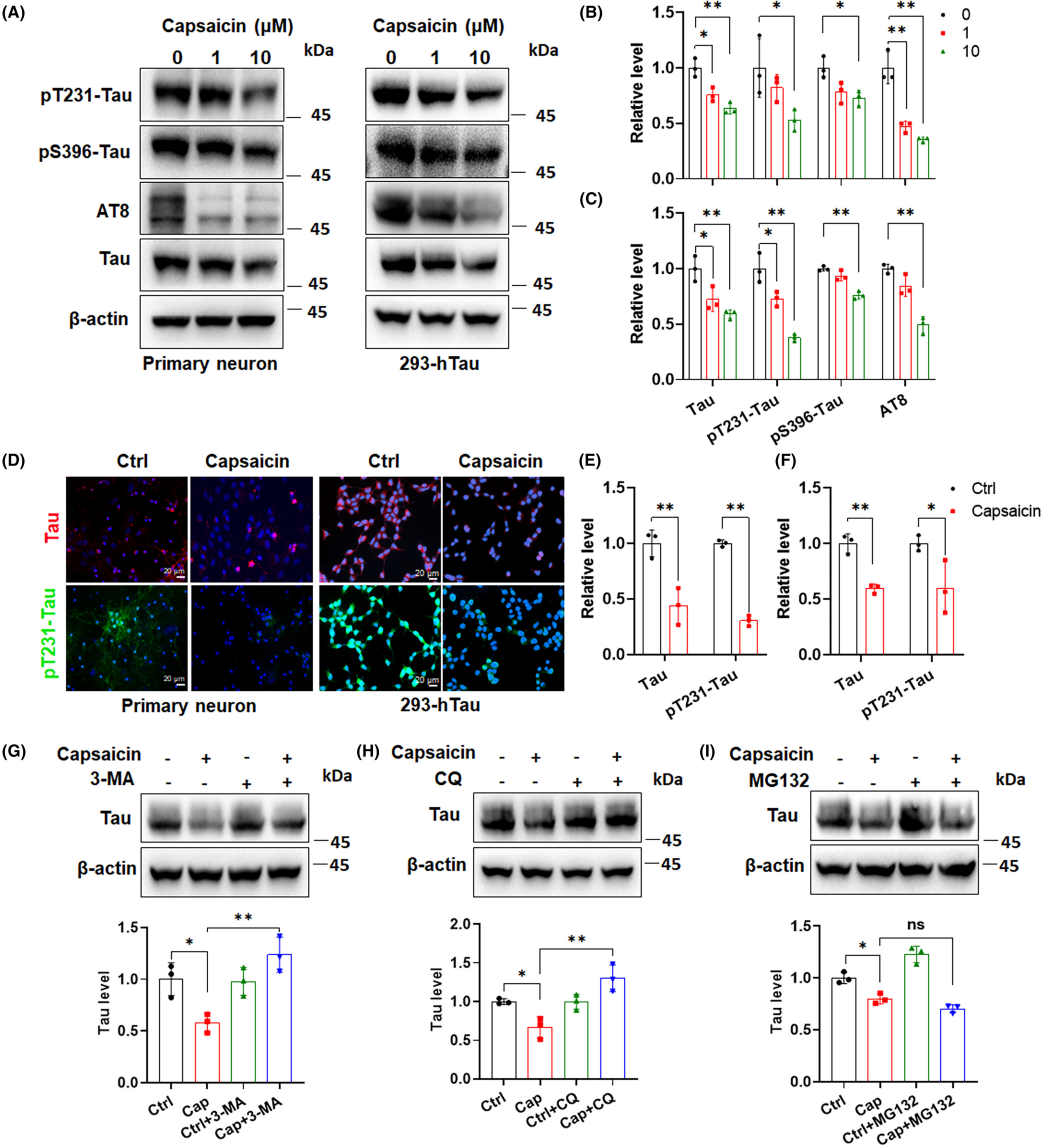
Capsaicin treatment‐induced tau degradation is dependent on the autophagy pathway. (A–C) Concentration‐dependent reduction of total tau and phosphorylated tau levels in primary neurons (A,B) and 293‐hTau cells (A,C) treated with capsaicin for 24 h. (D–F) Immunofluorescence analysis of total tau and pT231‐tau levels in primary neurons (D,E) and 293‐hTau cells (D,F) treated with 10 μM capsaicin for 24 h. (G–I) Verification of the involvement of the autophagy pathway rather than the proteasome pathway, in capsaicin treatment‐induced tau degradation in primary neurons. For 3‐MA treatment, neurons were treated with vehicle or capsaicin for 12 h and then co‐incubated with 5 mM of 3‐MA for 12 h (G). For CQ treatment, neurons were treated with vehicle or capsaicin for 15 h, and then CQ at 20 μM was added to cell media (H). For MG132 treatment, neurons were incubated with vehicle or capsaicin for 12 h, and MG132 was added at 10 μM to cell media for another 12 h. Tau levels were determined using immunoblot analysis. *p < 0.05, **p < 0.01, ns, not significant. One‐way ANOVA followed by Tukey's *post‐hoc* test in B–C and G–I and two‐tailed unpaired *t*‐test in E and F.

To better support the connection between tau degradation and autophagy enhancement in vivo, we determined levels of total tau and phosphorylated tau in primary neurons and 293‐hTau cells treated with different concentrations of capsaicin. In general, we observed a capsaicin concentration‐dependent decline in both total tau and phosphorylated tau at multiple sites (Thr231, Ser396, and Ser202/Thr205) in primary neurons (Figure 7A,B). 293‐hTau cells also showed identical trends of reductions in total and phosphorylated tau contents upon capsaicin treatment (Figure 7A,C). Since capsaicin at 10 μM consistently and robustly induced tau elimination in two cell models, we further checked this by immunostaining of total tau and pT231‐tau in cells treated with vehicle or capsaicin. Consistently, the results proved the efficacy of capsaicin treatment in clearing both endogenous and aberrantly accumulated tau in cells (Figure 7D–F). Next, we examined whether blocking the autophagy pathway ablated capsaicin‐induced tau degradation in primary neurons by applying 3‐methyladenine (3‐MA) or CQ. As shown in Figure 7G,H, inhibition of autophagy by either 3‐MA or CQ reversed capsaicin treatment‐induced tau downregulation. However, the application of MG132, a proteasome inhibitor, was incapable of doing this (Figure 7I). Similar results were found in 293‐hTau cells (Figure S2). Thus, neuronal tau degradation following capsaicin treatment is dependent on the activation of cellular autophagy rather than on the proteasome system.

### 3.7 TRPV1‐mediated AMPK/mTOR regulation is required for capsaicin treatment‐induced autophagy enhancement and tau degradation

**FIGURE 8:**
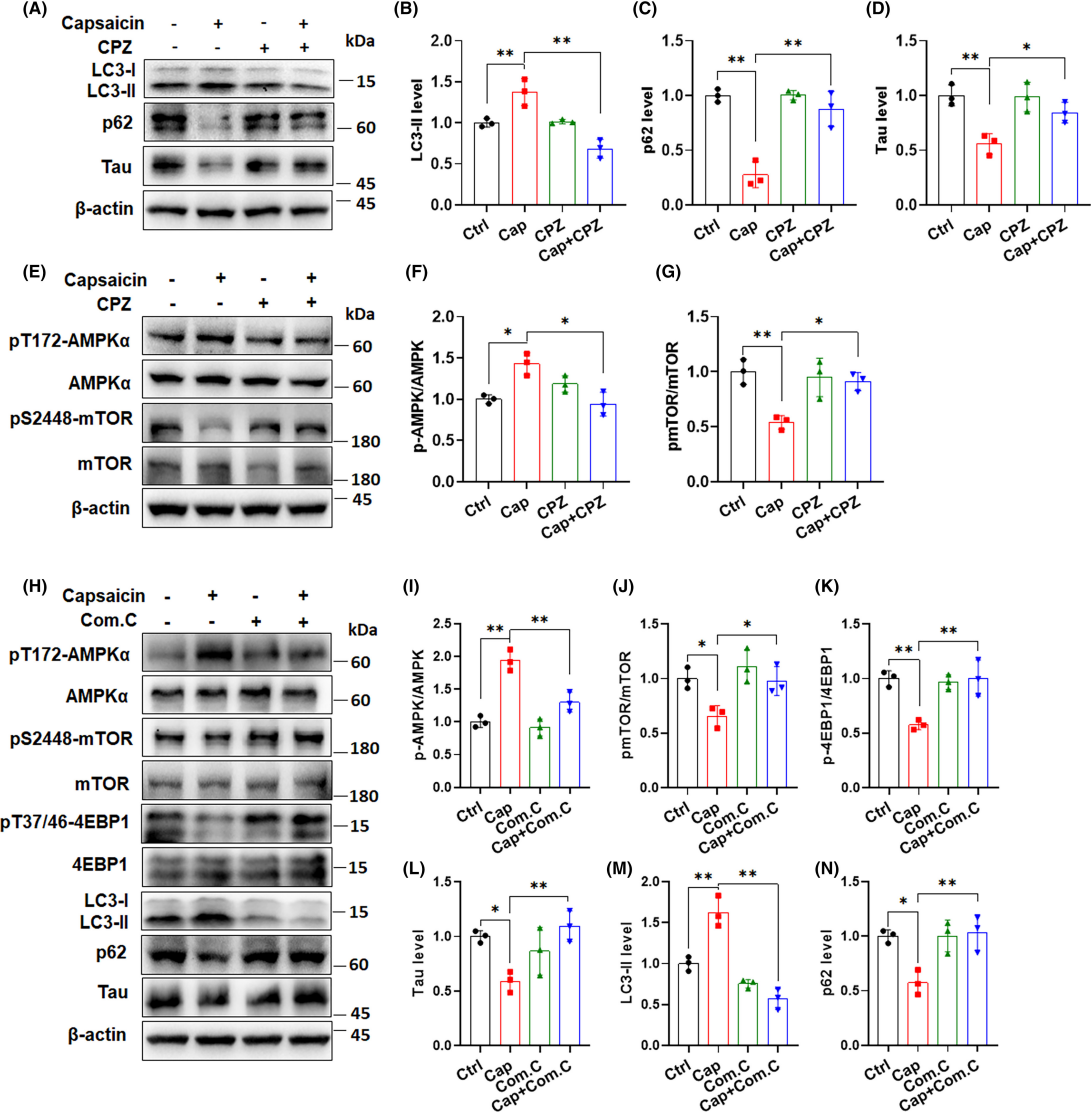
TRPV1‐mediated AMPK/mTOR regulation is required for capsaicin treatment‐induced autophagy enhancement and tau degradation. (A–D) 293‐hTau cells were treated with capsaicin (10 μM) alone, or together with the TRPV1 antagonist capsazepine (CPZ, 10 μM) for 24 h. Immunoblot analysis was applied to determine levels of autophagy markers LC3 (B) and p62 (C), and tau (D). (E–G) 293‐hTau cells were incubated with capsaicin alone (10 μM), or together with 10 μM of CPZ for 24 h, and the phosphorylation of AMPK (F) and mTOR (G) were analyzed by immunoblot analysis. (H–N) 293‐hTau cells were treated with 10 μM of capsaicin for 23.5 h, and the AMPK inhibitor Com.C (20 μM) or vehicle was added to cell media for half an hour before sample collection. The phosphorylation of AMPK (I), mTOR (J), and 4EBP1 (K), and changes in tau levels (L), and the autophagy markers LC3‐II (M) and p62 (N) were evaluated using immunoblot analysis. *p < 0.05, **p < 0.01. One‐way ANOVA followed by Tukey's *post‐hoc* test.

Having established a link between capsaicin‐induced tau elimination and autophagy enhancement, we wondered whether TRPV1, the endogenous receptor for capsaicin, participates in this process. To this end, we treated 293‐hTau cells and primary neurons with capsazepine (CPZ), a potent TRPV1 antagonist that competes with capsaicin and suppresses Ca^2+^ influx. CPZ treatment alone did not significantly affect basal autophagy and tau levels in 293‐hTau cells, as revealed by unchanged LC3‐II, p62, and total tau levels (Figure 8A). However, co‐treatment with CPZ and capsaicin led to a significant decrease in the generation of LC3‐II and partial recovery of p62 levels in 293‐hTau cells, implicating a decrease in autophagic function (Figure 8A–C). Correspondingly, the tau reduction caused by capsaicin treatment was reversed in 293‐hTau cells exposed to CPZ (Figure 8A,D). The abolishment of capsaicin‐induced autophagy enhancement and tau degradation by CPZ treatment in cells strongly supports the involvement of TRPV1 in mediating the effect of capsaicin on tau degradation. This dependency on TRPV1 was further confirmed in primary neurons subjected to capsaicin and CPZ treatment, in which the deactivation of TRPV1 fully blocked LC3‐II formation and maintained cellular p62 and tau levels (Figure S3A–D). Tau immunostaining in primary neurons treated with capsaicin alone or together with CPZ also presented a similar trend (Figure S3E,F). Therefore, we concluded that TRPV1 is indispensable for capsaicin‐induced autophagy activation and subsequent tau elimination in neurons.

**FIGURE 9:**
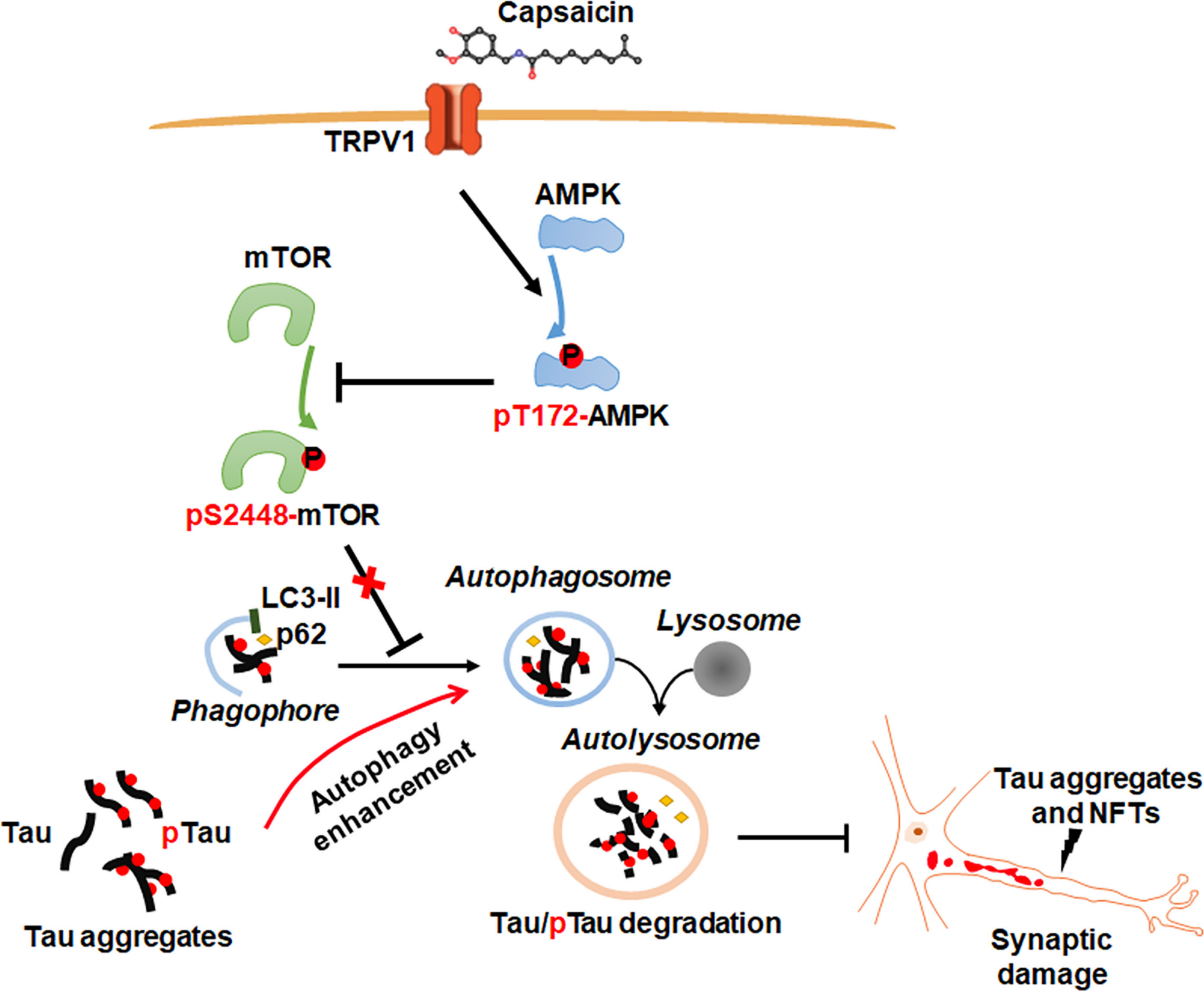
Schematic model of the effect of capsaicin‐mediated TRPV1 activation on autophagy function and tau degradation. Our study demonstrates that TRPV1 activation by capsaicin potentiates the autophagy pathway‐dependent degradation of both total and hyperphosphorylated tau proteins, leading to reduced accumulation of abnormal tau species and improved synaptic function in neurons and thus helping to rescue cognitive impairment caused by tauopathy. The TRPV1 activation‐induced autophagy enhancement is mediated by the AMPK/mTOR signaling pathway.

As in vivo experiments demonstrated that the AMPK/mTOR pathway in the mouse hippocampus was modulated by capsaicin treatment, we aimed to elucidate whether AMPK/mTOR signaling acts downstream of TRPV1 activation in regulating cellular autophagy and tau degradation. We first tested the activation of AMPK by capsaicin treatment in primary neurons and 293‐hTau cells. The phosphorylation of AMPK at Thr172 residues (pT172‐AMPK) increased in both cell models with the addition of capsaicin (Figure S4), indicating activated AMPK function by the compound. We then investigated the contribution of TRPV1 in modulating the AMPK/mTOR pathway. CPZ suppressed capsaicin treatment‐induced AMPK activation in 293‐hTau cells, thereby preserving mTOR phosphorylation at Ser2448 (Figure 8E–G). Similar changes in AMPK and mTOR phosphorylation could also be detected in primary neurons treated with capsaicin and CPZ (Figure S5). Therefore, TRPV1 activation is important for capsaicin‐induced AMPK activation and mTOR inhibition, which further corroborates the participation of TRPV1 in facilitating cellular autophagy. Next, we interrupted the AMPK activation by using its inhibitor compound C (Com.C) and studied whether this could affect cellular autophagy and tau degradation in 293‐hTau cells. It should be mentioned that we did not see a significant alteration in levels of AMPK/mTOR pathway‐related molecules and autophagy markers in cells incubated with Com.C alone (Figure 8H), probably due to the short treatment duration (0.5 h before sample collection). Yet we found that the addition of Com.C to 293‐hTau cells pretreated with capsaicin completely prevented AMPK phosphorylation at T172, leading to the subsequent restoration of mTOR activity, as exhibited by remarkable elevation of pS2448‐mTOR and pT37/46‐4EBP1 levels than samples treated with only capsaicin (Figure 8H–K). The decrease in tau levels caused by capsaicin was also reversed, together with LC3‐II formation and p62 reduction, after applying Com.C to 293‐hTau cells (Figure 8H,L–N). Furthermore, we proved the involvement of TRPV1/AMPK/mTOR cascade in mediating capsaicin‐induced autophagy enhancement and tau degradation by knocking down TRPV1 expression in 293‐hTau cells (Figure S6). These results provide convincing evidence that TRPV1 activation by capsaicin leads to autophagy‐mediated tau degradation by modulating the AMPK/mTOR pathway. To summarize the in vivo and in vitro data, we propose that stimulating TRPV1 activity improves neuronal autophagy via the AMPK/mTOR pathway. Autophagy enhancement promotes the degradation of aberrant tau protein and protects neuronal synapses, ultimately rescuing neuronal functions and cognitive impairment in AD mouse models (Figure 9).

## 4 DISCUSSION

Tau and Aβ pathologies represent two of the major targets for the development of disease‐modifying therapies for AD. The high spatio‐temporal correlation between tau pathology and neurodegeneration is the basis of disease staging and diagnosis.^46^ However, the intracellular nature of misfolded tau species and their potential propagation across neurons limit the efficacy of most anti‐tau antibodies.^47^ Thus, maintaining intraneuronal tau homeostasis by manipulating tau degradation serves as a promising approach to protect neurons against tauopathy. Herein, we demonstrate that TRPV1 activation affects neuronal tau elimination and synaptic functions by affecting autophagy using a mouse model overexpressing tau protein and two cell models. We further identified that the AMPK/mTOR pathway accounts for autophagy enhancement following TRPV1 activation. Capsaicin, a natural TRPV1 agonist, effectively alleviates synaptic damage and improves cognitive function via TRPV1‐dependent autophagy regulation.

TRPV1 is well known for its role in sensing nociceptive signals such as thermal stimuli, irritating chemicals, and acidic pH. Although originally identified in peripheral sensory neurons as a nociceptor, TRPV1 is also expressed in neurons and glial cells of the central nervous system,^48^ albeit its expression pattern in the brain needs thorough characterization. Some studies have proposed that hippocampal TRPV1 expression might be induced under pathological conditions such as Aβ treatment.^21^ In particular, the expression of TRPV1 in glial cells is thought to play a part in Aβ‐induced oxidative stress and neuroinflammation.^49^, ^50^ Another study also reported that TPRV1 knockout reduces Ca^2+^ overload in an AD mouse model and impedes both Aβ and tau pathologies.^24^ These results suggest a potentially detrimental role of TRPV1 in the pathogenesis of AD. In contrast with these findings, Balleza‐Tapia et al. showed that TRPV1 activation by capsaicin rescues synaptic transmission and gamma oscillations in mouse hippocampal slices exposed to toxic Aβ species.^21^ Our study confirmed the beneficial effects of TRPV1 activation on synaptic structure in a mouse model of tauopathy by showing restored PSD95 and MAP2 levels, as well as retained dendritic spine densities in mice treated with capsaicin, which may directly underlie the recovery of cognitive ability impaired by tau accumulation in mouse hippocampus. Analyses of tau transgenic mouse models and human AD brain tissues have disclosed that the expression of inflammation‐related genes is drastically enriched in microglia.^51^ A recent study reported that specifically overexpressing TRPV1 in microglia or applying the TRPV1 agonist capsaicin to APP/PS1 mice could improve Aβ‐induced energy metabolism deficiency, leading to not only restored microglial phagocytosis but also decreased secretion of pro‐inflammatory cytokines.^24^ We found that tau overexpression initiated the activation of microglia and astrocytes in the CA3 area, which could be repressed by capsaicin treatment. This might be attributed to reduced tau accumulation following TRPV1 activation or a direct impact of capsaicin on microglia and astrocytes.

TRPV1 activation by capsaicin or other chemicals has been shown to regulate cellular autophagy. For example, hypoxic injury induces the expression of TRPV1 in cardiomyocytes. This upregulation in turn protects cardiomyocytes from hypoxia by augmenting the autophagic flux through AMPK signaling.^52^ However, the upregulation of TRPV1 may also contribute to cell death by overactivating autophagy due to dysregulated Ca^2+^ levels and the downstream Ca^2+^/calmodulin‐dependent kinase kinase β (CaMKKβ)‐AMPK pathway.^53^ The difference suggests that the outcome of TRPV1‐induced autophagy may largely depend on the cellular context or the cell type. For instance, the activation of TRPV1 in microglia can facilitate the phagocytic degradation of both Aβ and α‐synuclein aggregates by enhancing autophagic function, resulting in improved neuronal functions and behavioral performance in AD or Parkinson's disease mouse models.^24^, ^25^, ^26^ Mechanistically, autophagy enhancement is mediated by Akt/mTOR and Ca^2+^‐CaMKKβ‐AMPK pathways, respectively. Similarly, Lin et al. described that TRPV1 activation antagonizes the microglia‐mediated inflammatory response in an ischemic injury model by regulating autophagy.^54^ Thus, TRPV1 activation‐induced autophagy is more likely to be beneficial in neurodegenerative diseases. The results from our study show that capsaicin treatment is able to enhance autophagic function in primary neurons and in the mouse hippocampus via TRPV1‐mediated AMPK/mTOR regulation. Furthermore, autophagy enhancement is directly responsible for the degradation of neuronal tau proteins in vitro and in vivo, as the inhibition of autophagy entirely reversed tau reduction in primary neurons treated with capsaicin. Besides, blocking TRPV1 or AMPK completely abolished the effect of capsaicin on autophagy and tau degradation, further confirming the central role of TRPV1 and AMPK in mediating autophagy enhancement in the brain. Tau overexpression has been revealed to activate mTOR activity by increasing cellular amino acid contents, which then inhibits autophagic function in neurons.^43^ We identified that capsaicin could potently inhibit mTOR activity in cells and mice, supporting TRPV1 activation as a potential intervention for neuronal damage in tauopathy.

The association between capsaicin and cognition is still under debate. Habitual spicy food consumption could affect the gray matter volumes of brain regions involved in capsaicin perception, indicating a potential link between capsaicin intake and brain structural changes.^55^ A cohort study based on adult Chinese population reported that high chili consumption is positively associated with lower cognitive status. In particular, the study pointed out that people with a daily chili intake above 50 g have approximately twice the risk of self‐reported memory problems than the non‐consumers.^56^ However, another group analyzed the correlation between capsaicin‐rich diet intake and AD‐related biomarkers and found that capsaicin consumption is correlated with higher cognitive ability, as measured by the Mini‐Mental State Examination. It is also negatively associated with total serum Aβ levels and Aβ40 levels.^16^ The same group further reported that people with more frequent spicy food consumption had better cognitive function in both normal and AD cohorts. Additionally, spicy food intake is inversely associated with the phospho‐tau/Aβ42 and total tau/Aβ42 ratios in the cerebrospinal fluid of non‐demented individuals.^15^ A follow‐up animal study reported that capsaicin could shift the processing of amyloid‐precursor protein toward the non‐amyloidogenic pathway by potentiating the maturation of the α‐secretase ADAM10, thereby reducing the generation of Aβ species in APP/PS1 mice.^17^ Interestingly, the study also showed that capsaicin treatment increased the content of PSD95, MAP2, and other synaptic markers, and reduced neuronal loss in APP/PS1 mice. In our tauopathy mouse model, both total and phosphorylated tau levels in neurons displayed a robust reduction after capsaicin intake, which may explain a recent research showing that capsaicin causes axonal ablation in sensory neurons by promoting microtubule depolymerization.^57^ Thus, our study identified a novel mechanism by which TRPV1 activation protects neuronal functions by mediating the clearance of highly accumulated tau protein.

Despite the reported beneficial effects of capsaicin against multiple AD‐related pathologies, the efficacy of capsaicin and other TRPV1 agonists as AD therapeutics should be carefully evaluated at both preclinical and clinical levels. Both body weight and swimming speed in the water maze were comparable between capsaicin‐treated and untreated mice, implying that capsaicin is safe and tolerable in mice when administered at the studied dose (0.0125%) and by the tested route of administration (food intake). The reported oral LD50 for capsaicin in male mice is 118.8 mg/kg.^58^ Compared with Wang et al., we used a higher capsaicin concentration in food (0.0125% vs. 0.01%), but our treatment duration was much shorter (9 weeks vs. 6 months),^17^ while both doses are well below the oral LD50 of capsaicin. Although we did not measure the concentration of capsaicin in the brain, a previous pharmacokinetics study showed that capsaicin can rapidly cross the blood–brain barrier when administered peripherally, although the metabolism of capsaicin in the brain appears to be slower than in peripheral organs.^59^ The excessive accumulation of capsaicin might cause unwanted damage to neurons, such as the impairment of synaptic transmission and neural network in hippocampus via TRPV1‐independent mechanisms.^21^, ^60^ High concentrations of capsaicin (e.g., 100 μM) may also damage axonal integrity by favoring microtubule depolymerization.^57^ In agreement with other studies, we used 10 μM of capsaicin to treat cells^21^, ^24^ and did not observe significant cell death or morphological changes. The PSD95 level in primary neurons was also comparable among cells treated with different concentrations of capsaicin (Figure S7). Given that levels of various biomarkers were almost identical between mice that received capsaicin alone and those treated with vehicle, we conclude that the concentrations of capsaicin used in our study are within the safe range and confer neuroprotection in the brain.

## 5 CONCLUSION

Overall, our study demonstrated that TRPV1 activation by capsaicin efficiently eliminates aberrantly accumulated tau protein in neurons through enhancing autophagic function mediated by the AMPK/mTOR pathway. The removal of excess tau improves synaptic function and cognitive performance in a mouse model of tauopathy. Our findings identify a novel molecular pathway through which TRPV1 affects neuronal functions in the brain. The data provide additional evidence to support the protective effect of capsaicin on neuronal function and cognition, and suggest that TRPV1 could be a potential disease‐modifying target for the intervention of tauopathies.

## AUTHOR CONTRIBUTIONS

THL and TZ conceived this project, designed experiments, analyzed the data, and wrote the manuscript. YT and XZ performed experiments and analyzed the data. DC and RL provided advice on animal experiments. LH, YM, XS, MZ, QW, LW, and XZ offered technical assistance. WT helped with advice on the experiments and manuscript editing. All authors discussed the results and contributed to the final manuscript.

## CONFLICT OF INTEREST STATEMENT

The authors declare no competing interests.

## Supporting information


Data S1.

## Data Availability

The datasets used and/or analyzed during the current study are available from the corresponding author upon reasonable request.

## References

[1] Wang Y , Mandelkow E . Tau in physiology and pathology. Nat Rev Neurosci. 2016;17(1):5‐21. doi:10.1038/nrn.2015.1 26631930

[2] Iwata M , Watanabe S , Yamane A , Miyasaka T , Misonou H . Regulatory mechanisms for the axonal localization of tau protein in neurons. Mol Biol Cell. 2019;30(19):2441‐2457. doi:10.1091/mbc.E19-03-0183 31364926 PMC6743362

[3] Rawat P , Sehar U , Bisht J , Selman A , Culberson J , Reddy PH . Phosphorylated tau in Alzheimer's disease and other tauopathies. Int J Mol Sci. 2022;23(21):12841. doi:10.3390/ijms232112841 36361631 PMC9654278

[4] Neddens J , Temmel M , Flunkert S , et al. Phosphorylation of different tau sites during progression of Alzheimer's disease. Acta Neuropathol Commun. 2018;6(1):52. doi:10.1186/s40478-018-0557-6 29958544 PMC6027763

[5] Zempel H , Mandelkow E . Lost after translation: missorting of tau protein and consequences for Alzheimer disease. Trends Neurosci. 2014;37(12):721‐732. doi:10.1016/j.tins.2014.08.004 25223701

[6] Young ZT , Mok SA , Gestwicki JE . Therapeutic Strategies for Restoring Tau Homeostasis. Cold Spring Harb Perspect Med. 2018;8(1):a024612. doi:10.1101/cshperspect.a024612 28159830 PMC5540800

[7] Lee MJ , Lee JH , Rubinsztein DC . Tau degradation: the ubiquitin‐proteasome system versus the autophagy‐lysosome system. Prog Neurobiol. 2013;105:49‐59. doi:10.1016/j.pneurobio.2013.03.001 23528736

[8] Jiang S , Bhaskar K . Degradation and transmission of tau by autophagic‐Endolysosomal networks and potential therapeutic targets for tauopathy. Front Mol Neurosci. 2020;13:586731. doi:10.3389/fnmol.2020.586731 33177989 PMC7596180

[9] Piras A , Collin L , Gruninger F , Graff C , Ronnback A . Autophagic and lysosomal defects in human tauopathies: analysis of post‐mortem brain from patients with familial Alzheimer disease, corticobasal degeneration and progressive supranuclear palsy. Acta Neuropathol Commun. 2016;4:22. doi:10.1186/s40478-016-0292-9 26936765 PMC4774096

[10] Nixon RA , Wegiel J , Kumar A , et al. Extensive involvement of autophagy in Alzheimer disease: an immuno‐electron microscopy study. J Neuropathol Exp Neurol. 2005;64(2):113‐122. doi:10.1093/jnen/64.2.113 15751225

[11] Malik BR , Maddison DC , Smith GA , Peters OM . Autophagic and endo‐lysosomal dysfunction in neurodegenerative disease. Mol Brain. 2019;12(1):100. doi:10.1186/s13041-019-0504-x 31783880 PMC6884906

[12] Schaeffer V , Lavenir I , Ozcelik S , Tolnay M , Winkler DT , Goedert M . Stimulation of autophagy reduces neurodegeneration in a mouse model of human tauopathy. Brain. 2012;135(Pt 7):2169‐2177. doi:10.1093/brain/aws143 22689910 PMC3381726

[13] Rodriguez‐Navarro JA , Rodriguez L , Casarejos MJ , et al. Trehalose ameliorates dopaminergic and tau pathology in parkin deleted/tau overexpressing mice through autophagy activation. Neurobiol Dis. 2010;39(3):423‐438. doi:10.1016/j.nbd.2010.05.014 20546895

[14] Kruger U , Wang Y , Kumar S , Mandelkow EM . Autophagic degradation of tau in primary neurons and its enhancement by trehalose. Neurobiol Aging. 2012;33(10):2291‐2305. doi:10.1016/j.neurobiolaging.2011.11.009 22169203

[15] Tian DY , Wang J , Sun BL , et al. Spicy food consumption is associated with cognition and cerebrospinal fluid biomarkers of Alzheimer disease. Chin Med J (Engl). 2020;134(2):173‐177. doi:10.1097/CM9.0000000000001318 33443937 PMC7817283

[16] Liu CH , Bu XL , Wang J , et al. The associations between a capsaicin‐rich diet and blood amyloid‐beta levels and cognitive function. J Alzheimers Dis. 2016;52(3):1081‐1088. doi:10.3233/JAD-151079 27079706

[17] Wang J , Sun BL , Xiang Y , et al. Capsaicin consumption reduces brain amyloid‐beta generation and attenuates Alzheimer's disease‐type pathology and cognitive deficits in APP/PS1 mice. Transl Psychiatry. 2020;10(1):230. doi:10.1038/s41398-020-00918-y 32661266 PMC7359297

[18] Bevan S , Quallo T , Andersson DA . Trpv1. Handb Exp Pharmacol. 2014;222:207‐245. doi:10.1007/978-3-642-54215-2_9 24756708

[19] Imler E , Zinsmaier KE . TRPV1 channels: not so inactive on the ER. Neuron. 2014;84(4):659‐661. doi:10.1016/j.neuron.2014.10.052 25459405

[20] Du Y , Fu M , Huang Z , et al. TRPV1 activation alleviates cognitive and synaptic plasticity impairments through inhibiting AMPAR endocytosis in APP23/PS45 mouse model of Alzheimer's disease. Aging Cell. 2020;19(3):e13113. doi:10.1111/acel.13113 32061032 PMC7059138

[21] Balleza‐Tapia H , Crux S , Andrade‐Talavera Y , et al. TrpV1 receptor activation rescues neuronal function and network gamma oscillations from Abeta‐induced impairment in mouse hippocampus in vitro. Elife. 2018;7:e37703. doi:10.7554/eLife.37703 30417826 PMC6281315

[22] Grimm MOW , Blumel T , Lauer AA , et al. The impact of capsaicinoids on APP processing in Alzheimer's disease in SH‐SY5Y cells. Sci Rep. 2020;10(1):9164. doi:10.1038/s41598-020-66009-6 32514053 PMC7280252

[23] Kim J , Lee S , Kim J , et al. Ca^2+^−permeable TRPV1 pain receptor knockout rescues memory deficits and reduces amyloid‐beta and tau in a mouse model of Alzheimer's disease. Hum Mol Genet. 2020;29(2):228‐237. doi:10.1093/hmg/ddz276 31814000

[24] Lu J , Zhou W , Dou F , Wang C , Yu Z . TRPV1 sustains microglial metabolic reprogramming in Alzheimer's disease. EMBO Rep. 2021;22(6):e52013. doi:10.15252/embr.202052013 33998138 PMC8183394

[25] Yuan J , Liu H , Zhang H , Wang T , Zheng Q , Li Z . Controlled activation of TRPV1 channels on microglia to boost their autophagy for clearance of alpha‐synuclein and enhance therapy of Parkinson's disease. Adv Mater. 2022;34(11):e2108435. doi:10.1002/adma.202108435 35023596

[26] Lu J , Wang C , Cheng X , et al. A breakdown in microglial metabolic reprogramming causes internalization dysfunction of alpha‐synuclein in a mouse model of Parkinson's disease. J Neuroinflammation. 2022;19(1):113. doi:10.1186/s12974-022-02484-0 35599331 PMC9124408

[27] Yin Y , Gao D , Wang Y , et al. Tau accumulation induces synaptic impairment and memory deficit by calcineurin‐mediated inactivation of nuclear CaMKIV/CREB signaling. Proc Natl Acad Sci USA. 2016;113(26):E3773‐E3781. doi:10.1073/pnas.1604519113 27298345 PMC4932970

[28] Bromley‐Brits K , Deng Y , Song W . Morris water maze test for learning and memory deficits in Alzheimer's disease model mice. J Vis Exp. 2011;(53):e2920. doi:10.3791/2920 PMC3347885 21808223

[29] Wang L , Shui X , Zhang M , et al. MiR‐191‐5p attenuates tau phosphorylation, Abeta generation, and neuronal cell death by regulating death‐associated protein kinase 1. ACS Chem Nerosci. 2022;13(24):3554‐3566. doi:10.1021/acschemneuro.2c00423 36454178

[30] Zhang T , Xia Y , Hu L , et al. Death‐associated protein kinase 1 mediates Abeta42 aggregation‐induced neuronal apoptosis and tau dysregulation in Alzheimer's disease. Int J Biol Sci. 2022;18(2):693‐706. doi:10.7150/ijbs.66760 35002518 PMC8741852

[31] Chen D , Lan G , Li R , et al. Melatonin ameliorates tau‐related pathology via the miR‐504‐3p and CDK5 axis in Alzheimer's disease. Transl Neurodegener. 2022;11(1):27. doi:10.1186/s40035-022-00302-4 35527277 PMC9082841

[32] Chen D , Mei Y , Kim N , et al. Melatonin directly binds and inhibits death‐associated protein kinase 1 function in Alzheimer's disease. J Pineal Res. 2020;69(2):e12665. doi:10.1111/jpi.12665 32358852 PMC7890046

[33] Schwalbe M , Kadavath H , Biernat J , et al. Structural impact of tau phosphorylation at threonine 231. Structure. 2015;23(8):1448‐1458. doi:10.1016/j.str.2015.06.002 26165593

[34] Suarez‐Calvet M , Karikari TK , Ashton NJ , et al. Novel tau biomarkers phosphorylated at T181, T217 or T231 rise in the initial stages of the preclinical Alzheimer's continuum when only subtle changes in Abeta pathology are detected. EMBO Mol Med. 2020;12(12):e12921. doi:10.15252/emmm.202012921 33169916 PMC7721364

[35] Wu M , Zhang M , Yin X , et al. The role of pathological tau in synaptic dysfunction in Alzheimer's diseases. Transl Neurodegener. 2021;10(1):45. doi:10.1186/s40035-021-00270-1 34753506 PMC8579533

[36] Cane M , Maco B , Knott G , Holtmaat A . The relationship between PSD‐95 clustering and spine stability in vivo. J Neurosci. 2014;34(6):2075‐2086. doi:10.1523/JNEUROSCI.3353-13.2014 24501349 PMC6608529

[37] Li B , Chohan MO , Grundke‐Iqbal I , Iqbal K . Disruption of microtubule network by Alzheimer abnormally hyperphosphorylated tau. Acta Neuropathol. 2007;113(5):501‐511. doi:10.1007/s00401-007-0207-8 17372746 PMC3191942

[38] Didonna A . Tau at the interface between neurodegeneration and neuroinflammation. Genes Immun. 2020;21(5):288‐300. doi:10.1038/s41435-020-00113-5 33011744

[39] Morales I , Jimenez JM , Mancilla M , Maccioni RB . Tau oligomers and fibrils induce activation of microglial cells. J Alzheimers Dis. 2013;37(4):849‐856. doi:10.3233/JAD-131843 23948931

[40] Ising C , Venegas C , Zhang S , et al. NLRP3 inflammasome activation drives tau pathology. Nature. 2019;575(7784):669‐673. doi:10.1038/s41586-019-1769-z 31748742 PMC7324015

[41] Wang C , Fan L , Khawaja RR , et al. Microglial NF‐kappaB drives tau spreading and toxicity in a mouse model of tauopathy. Nat Commun. 2022;13(1):1969. doi:10.1038/s41467-022-29552-6 35413950 PMC9005658

[42] Chang HY , Sang TK , Chiang AS . Untangling the tauopathy for Alzheimer's disease and parkinsonism. J Biomed Sci. 2018;25(1):54. doi:10.1186/s12929-018-0457-x 29991349 PMC6038292

[43] Li MZ , Liu EJ , Zhou QZ , et al. Intracellular accumulation of tau inhibits autophagosome formation by activating TIA1‐amino acid‐mTORC1 signaling. Mil Med Res. 2022;9(1):38. doi:10.1186/s40779-022-00396-x 35799293 PMC9264508

[44] Querfurth H , Lee HK . Mammalian/mechanistic target of rapamycin (mTOR) complexes in neurodegeneration. Mol Neurodegener. 2021;16(1):44. doi:10.1186/s13024-021-00428-5 34215308 PMC8252260

[45] Chen X , Li Y , Wang C , et al. Promoting tau secretion and propagation by hyperactive p300/CBP via autophagy‐lysosomal pathway in tauopathy. Mol Neurodegener. 2020;15(1):2. doi:10.1186/s13024-019-0354-0 31906970 PMC6945522

[46] Braak H , Alafuzoff I , Arzberger T , Kretzschmar H , Del Tredici K . Staging of Alzheimer disease‐associated neurofibrillary pathology using paraffin sections and immunocytochemistry. Acta Neuropathol. 2006;112(4):389‐404. doi:10.1007/s00401-006-0127-z 16906426 PMC3906709

[47] Sandusky‐Beltran LA , Sigurdsson EM . Tau immunotherapies: lessons learned, current status and future considerations. Neuropharmacology. 2020;175:108104. doi:10.1016/j.neuropharm.2020.108104 32360477 PMC7492435

[48] Duitama M , Vargas‐Lopez V , Casas Z , Albarracin SL , Sutachan JJ , Torres YP . TRP channels role in pain associated with neurodegenerative diseases. Front Neurosci. 2020;14:782. doi:10.3389/fnins.2020.00782 32848557 PMC7417429

[49] Schilling T , Eder C . Amyloid‐beta‐induced reactive oxygen species production and priming are differentially regulated by ion channels in microglia. J Cell Physiol. 2011;226(12):3295‐3302. doi:10.1002/jcp.22675 21321937

[50] Benito C , Tolon RM , Castillo AI , et al. Beta‐amyloid exacerbates inflammation in astrocytes lacking fatty acid amide hydrolase through a mechanism involving PPAR‐alpha, PPAR‐gamma and TRPV1, but not CB(1) or CB(2) receptors. Br J Pharmacol. 2012;166(4):1474‐1489. doi:10.1111/j.1476-5381.2012.01889.x 22321194 PMC3417461

[51] Rexach JE , Polioudakis D , Yin A , et al. Tau pathology drives dementia risk‐associated gene networks toward chronic inflammatory states and immunosuppression. Cell Rep. 2020;33(7):108398. doi:10.1016/j.celrep.2020.108398 33207193 PMC7842189

[52] Wei J , Lin J , Zhang J , et al. TRPV1 activation mitigates hypoxic injury in mouse cardiomyocytes by inducing autophagy through the AMPK signaling pathway. Am J Physiol Cell Physiol. 2020;318(5):C1018‐C1029. doi:10.1152/ajpcell.00161.2019 32293932

[53] Chen M , Dong X , Deng H , et al. Targeting TRPV1‐mediated autophagy attenuates nitrogen mustard‐induced dermal toxicity. Signal Transduct Target Ther. 2021;6(1):29. doi:10.1038/s41392-020-00389-z 33487631 PMC7829253

[54] Lin Y , Huang T , Shen W , et al. TRPV1 suppressed NLRP3 through regulating autophagy in microglia after ischemia‐reperfusion injury. J Mol Neurosci. 2022;72(4):792‐801. doi:10.1007/s12031-021-01935-2 35041191

[55] Han P , Su T , Chen H , Hummel T . Regional brain morphology of the primary somatosensory cortex correlates with spicy food consumption and capsaicin sensitivity. Nutr Neurosci. 2023;26(3):208‐216. doi:10.1080/1028415X.2022.2031495 35156563

[56] Shi Z , El‐Obeid T , Riley M , Li M , Page A , Liu J . High chili intake and cognitive function among 4582 adults: an open cohort study over 15 years. Nutrients. 2019;11(5):1183. doi:10.3390/nu11051183 31137805 PMC6566199

[57] Arora V , Li T , Kumari S , Wang S , Asgar J , Chung MK . Capsaicin‐induced depolymerization of axonal microtubules mediates analgesia for trigeminal neuropathic pain. Pain. 2022;163(8):1479‐1488. doi:10.1097/j.pain.0000000000002529 34724681 PMC9046530

[58] Saito A , Yamamoto M . Acute oral toxicity of capsaicin in mice and rats. J Toxicol Sci. 1996;21(3):195‐200. doi:10.2131/jts.21.3_195 8887888

[59] Rollyson WD , Stover CA , Brown KC , et al. Bioavailability of capsaicin and its implications for drug delivery. J Control Release. 2014;196:96‐105. doi:10.1016/j.jconrel.2014.09.027 25307998 PMC4267963

[60] Benninger F , Freund TF , Hajos N . Control of excitatory synaptic transmission by capsaicin is unaltered in TRPV1 vanilloid receptor knockout mice. Neurochem Int. 2008;52(1–2):89‐94. doi:10.1016/j.neuint.2007.06.008 17651868 PMC2194163

