# Supplementary material for: Activation of transient receptor potential vanilloid 1 ameliorates tau accumulation‐induced synaptic damage and cognitive dysfunction via autophagy enhancement
Source: CNS Neurosci Ther. 2023 Aug 29;30(3):e14432. doi: 10.1111/cns.14432 (PMC10916438; doi:10.1111/cns.14432)
Supplement: Supplementary file 1 — Data S1. [file CNS-30-e14432-s001.pdf]

Supplementary data

Activation of transient receptor potential vanilloid 1 ameliorates Tau accumulation induced synaptic damage and cognitive dysfunction via autophagy enhancement

Tao Zhang[1 #], Yuan Tian[1 #], Xiaoqing Zheng[1], Ruomeng Li[1], Li Hu[1], Xindong Shui[1], Yingxue Mei[1], Quling Wang[1], Mi Zhang[1], Xiuzhi Zheng[1], Long Wang[1], Dongmei Chen[1], Wucheng Tao[1,2], Tae Ho Lee[1 *]

[1] Fujian Key Laboratory of Translational Research in Cancer and Neurodegenerative Diseases,
School of Basic Medical Sciences,
Fujian Medical University,
Fuzhou, Fujian, 350122, China
[2] Key Laboratory of Brain Aging and Neurodegenerative Diseases,
School of Basic Medical Sciences,
Fujian Medical University,
Fuzhou, Fujian, 350122, China.

[#] These authors contributed equally to this work.

[*] Correspondence: Tae Ho Lee, Fujian Medical University, 1 Xuefu North Road, Fuzhou, Fujian 350122, China; E-mail: tlee0813@fjmu.edu.cn; Tel.: +86-591-2286-2498; Fax: +86-591-2286-2320.

Running head: TRPV1 promotes tau degradation via autophagy

Supplementary Table S1. Information about antibodies used in the study.

Antibody	Dilution	Source	Identifier
Rabbit anti-pT231-Tau	1: 1000 (IB)<br>1: 50 (IF)	Abcam	ab151559
Rabbit anti-pS396-Tau	1:1000 (IB)	Anaspec	AS-54977
Mouse anti-pS202/T205-Tau (AT8)	1:1000 (IB)	Invitrogen	MN1020
Mouse anti-Tau (HT7)	1:10000 (IB)<br>1:500 (IF)	Invitrogen	MN1000
Mouse anti-Tau-5	1:500 (IB)	Invitrogen	AHB0042
Rabbit anti-PSD95	1:1000 (IB)	Abcam	ab18258
Rabbit anti-MAP2	1:50 (IF)	Cell Signaling Technology	4542S
Rabbit anti-GFAP	1:500 (IF)	Abcam	ab7260
Rabbit anti-IBA1	1:500 (IF)	Abcam	ab178847
Rabbit anti-LC3	1:2000 (IB)<br>1:100 (IF)	Cell Signaling Technology	12741S
Rabbit anti-p62	1:1000 (IB)	Cell Signaling Technology	5114S
Rabbit anti-mTOR	1:1000 (IB)	Cell Signaling Technology	2972S
Rabbit anti-pS2448-mTOR	1:1000 (IB)	Cell Signaling Technology	5536P
Rabbit anti-AMPKα	1:1000 (IB)	Cell Signaling Technology	5831S
Rabbit anti-pT172-AMPKα	1:1000 (IB)	Cell Signaling Technology	2535S
Rabbit anti-pT37/46-4EBP1	1:1000 (IB)	Cell Signaling Technology	2855P
Rabbit anti-4EBP1	1:1000 (IB)	Cell Signaling Technology	9644S
Mouse anti-TRPV1	1:1000 (IB)	Proteintech	66983-1-Ig
Mouse anti-β-actin	1:40000 (IB)	Sigma	A5441
HRP-conjugated goat anti-rabbit secondary antibody	1:10000	Bio-rad	1706515
HRP-conjugated goat anti-mouse secondary antibody	1:10000	Bio-rad	1706516
Alexa Fluor 488 goat anti-rabbit secondary antibody	1:400	Invitrogen	A11034
Alexa Fluor 488 goat anti-mouse secondary antibody	1:400	Invitrogen	A11029
Alexa Fluor 546 goat anti-rabbit secondary antibody	1:400	Invitrogen	A11035
Alexa Fluor 546 goat anti-mouse secondary antibody	1:400	Invitrogen	A11030

IB, immunoblot analysis; IF, immunofluorescence imaging analysis.

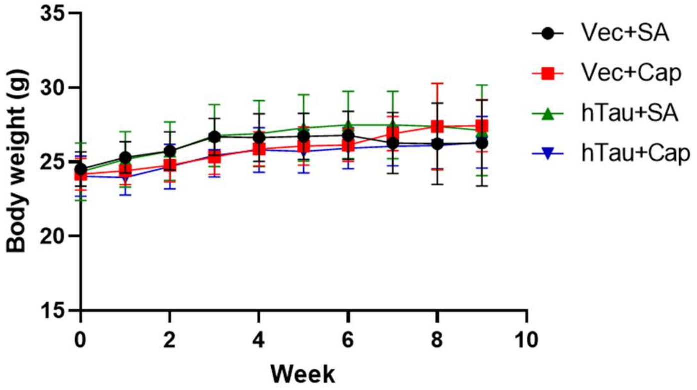

Supplementary Figure S1. Body weight of four groups of mice receiving either normal diet or capsaicin diet over the treatment duration. No significant change was observed among all mice.

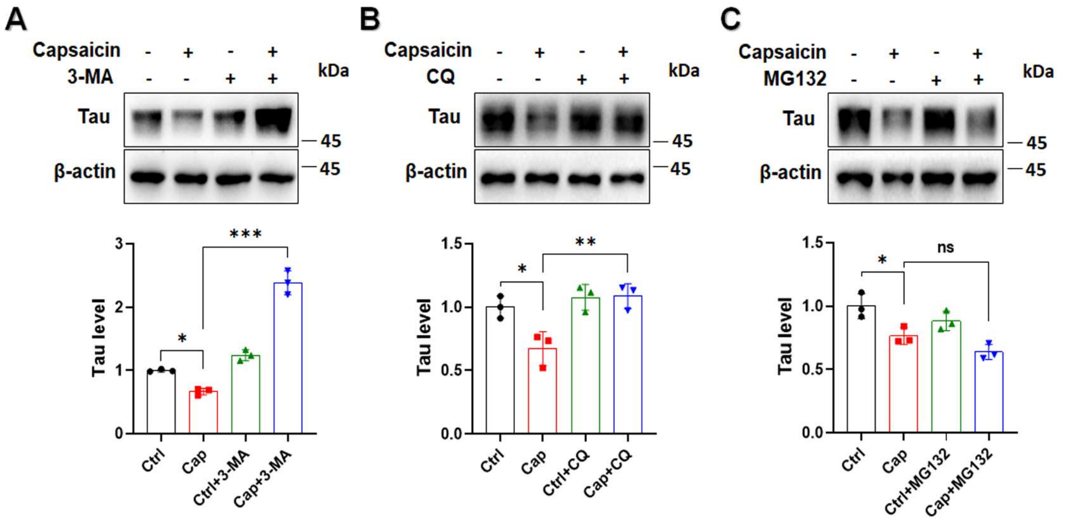

Supplementary Figure S2. Capsaicin treatment-induced tau degradation is dependent on the autophagy pathway in 293-hTau cells. The 293-hTau cells were treated in the same manner as primary neurons described in the main text, and the tau levels were compared to verify whether the tau degradation is mediated by the autophagy pathway or the proteasome pathway. *p < 0.05, **p < 0.01, ***p < 0.001, ns, not significant. One-way ANOVA followed by Tukey's *post-hoc* test.

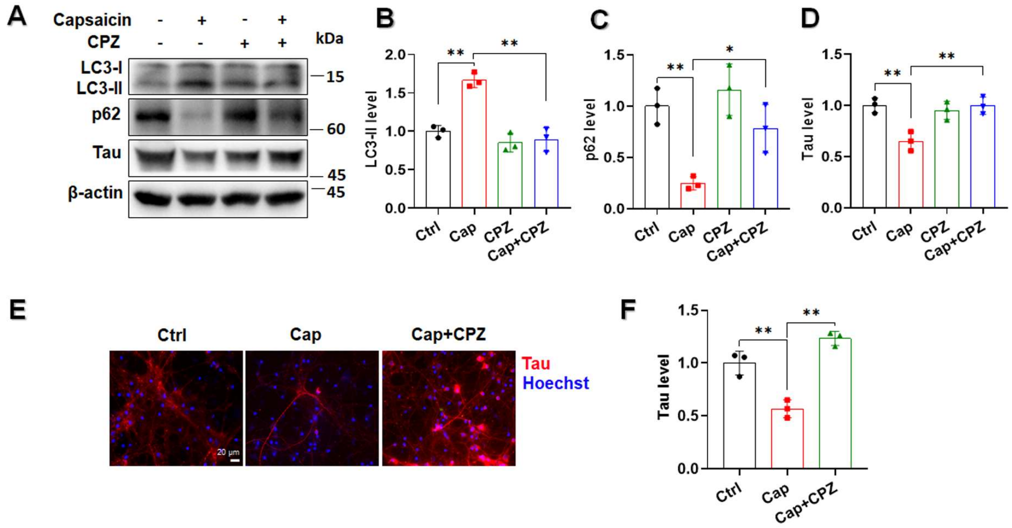

Supplementary Figure S3. TRPV1 is required for capsaicin treatment-induced autophagy enhancement and tau degradation in neurons. Mouse primary neurons were treated with capsaicin (10 μM) alone, or together with the TRPV1 antagonist capsazepine (CPZ, 10 μM) for 24 h. Immunoblot analysis was applied to determine levels of autophagy markers LC3 (B) and p62 (C), and tau (D). (E-F) Immunostaining of total tau level in primary neurons treated with capsaicin alone, or capsaicin plus CPZ for 24 h. *$p < 0.05$, **$p < 0.01$. One-way ANOVA followed by Tukey's *post-hoc* test.

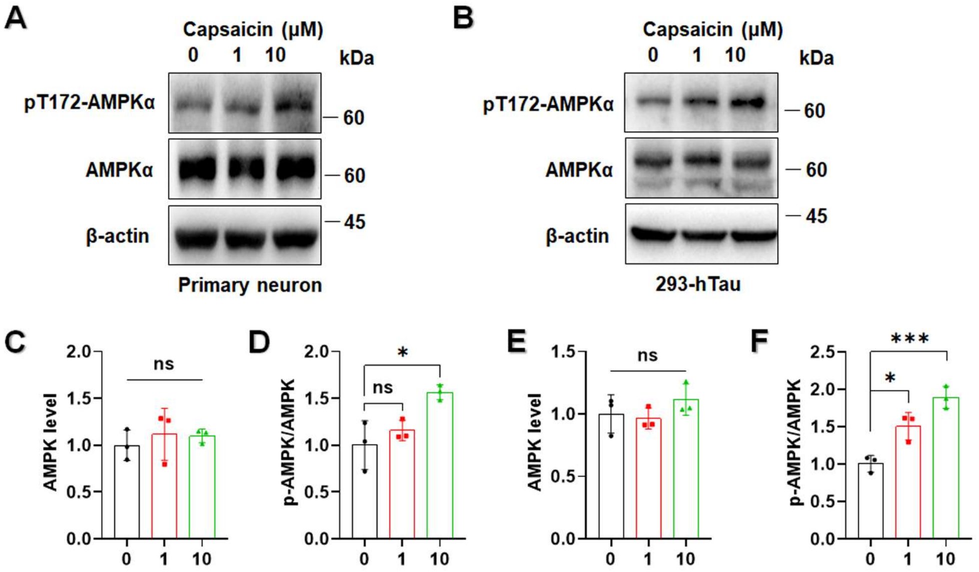

Supplementary Figure S4. Capsaicin treatment facilitates AMPKα phosphorylation in a concentration-dependent manner. Primary neurons or 293-hTau cells were treated with different concentrations of capsaicin for 24 h, and the phosphorylation of AMPKα was detected

AMPKα phosphorylation at Thr172 residues (pT172-AMPKα) increases with the elevation of

post-hoc test.

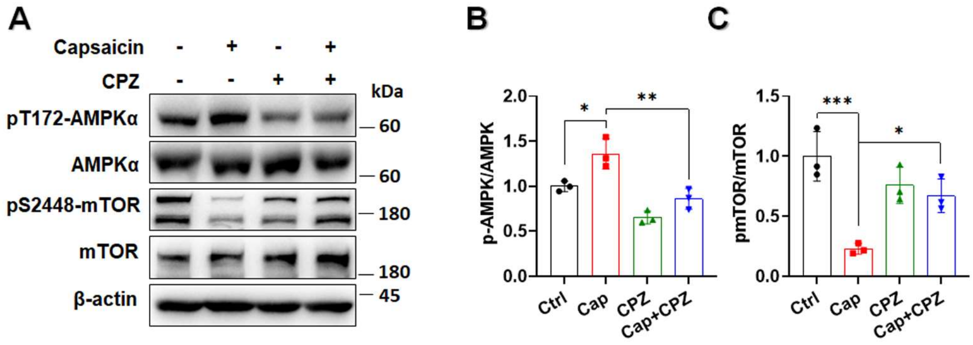

Supplementary Figure S5. Capsaicin-induced AMPKα phosphorylation is dependent on TPRV1 in primary neurons. Primary neurons were treated with capsaicin (10 μM) alone, or together with TRPV1 antagonist capsazepine (CPZ, 10 μM) for 24 h, and changes in AMPK and mTOR phosphorylation were determined by immunoblot analysis. The result shows that capsaicin induced AMPK activation and mTOR inhibition could be completely suppressed by TRPV1 blockade. *$p < 0.05$, **$p < 0.01$, ***$p < 0.001$. One-way ANOVA followed by Tukey's *post-hoc* test.

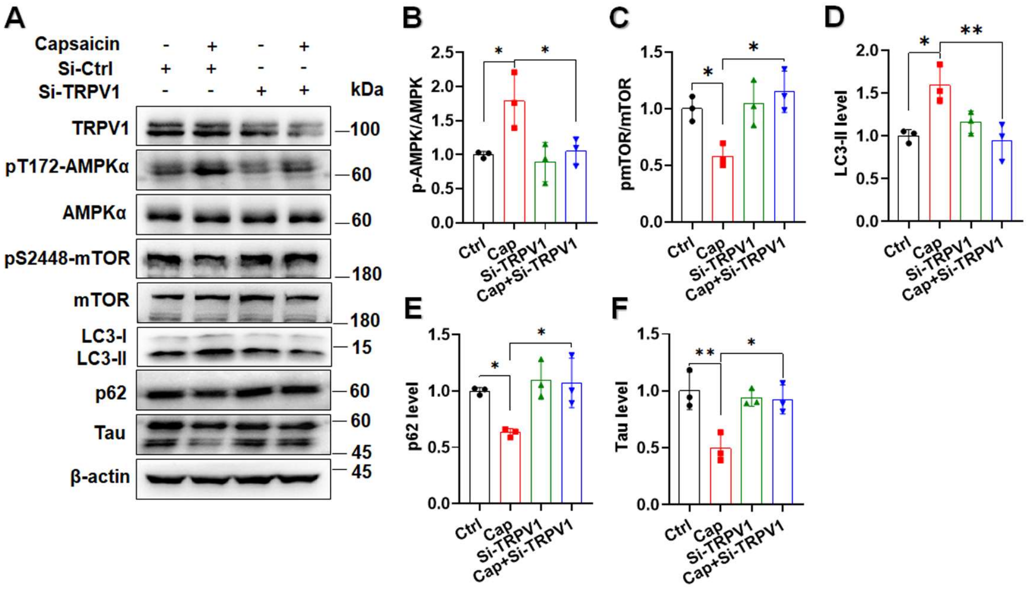

Supplementary Figure S6. TRPV1 knockdown abolishes the effect of capsaicin on cellular autophagy and tau degradation. 293-hTau cells were transfected with control SiRNA or human TRPV1 SiRNA (50 nM). One day post transfection, cells were treated with vehicle or capsaicin (10 μM) for 24 h. The expression of TRPV1 was first determined to prove the efficiency of SiRNA knockdown. Moreover, the phosphorylation of AMPK (B) and mTOR (C), the expression of autophagy markers LC3 (D) and p62 (E), and the total tau levels (F) in different samples were quantified. *$p < 0.05$, **$p < 0.01$. One-way ANOVA followed by Tukey's *post-hoc* test.

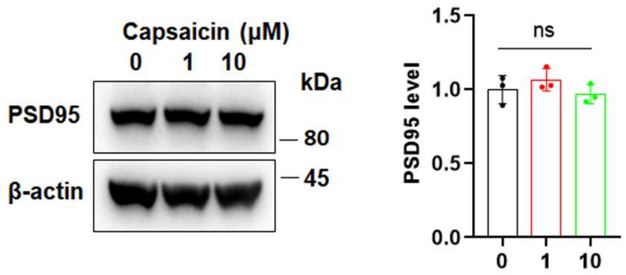

Supplementary Figure S7. Capsaicin does not reduce neuronal PSD95 level. Primary neurons were treated with different concentrations of capsaicin for 24 h, and the PSD95 content was detected using immunoblot analysis. The result indicates that under the present concentrations, capsaicin treatment does not alter PSD95 expression in neurons. ns, not significant. One-way ANOVA followed by Tukey's *post-hoc* test.
